# Advancing risk stratification in kidney transplantation: integrating HLA-derived T-cell epitope and B-cell epitope matching algorithms for enhanced predictive accuracy of HLA compatibility

**DOI:** 10.3389/fimmu.2025.1548934

**Published:** 2025-02-11

**Authors:** Matthias Niemann, Benedict M. Matern, Gaurav Gupta, Bekir Tanriover, Fabian Halleck, Klemens Budde, Eric Spierings

**Affiliations:** ^1^ Research and Development, PIRCHE AG, Berlin, Germany; ^2^ Department of Nephrology and Medical Intensive Care, Charité Universitätsmedizin Berlin, Berlin, Germany; ^3^ Center for Translational Immunology, University Medical Center, Utrecht, Netherlands; ^4^ Department of Internal Medicine, Virginia Commonwealth University, Richmond, VA, United States; ^5^ Division of Nephrology, The University of Arizona, Tucson, AZ, United States; ^6^ Central Diagnostic Laboratory, University Medical Center, Utrecht, Netherlands

**Keywords:** molecular matching, XGBoost, Snow, PIRCHE, clinical prediction model, kidney transplantation, epitope matching

## Abstract

**Introduction:**

The immune-mediated rejection of transplanted organs is a complex interplay between T cells and B cells, where the recognition of HLA-derived epitopes plays a crucial role. Several algorithms of molecular compatibility have been suggested, each focusing on a specific aspect of epitope immunogenicity.

**Methods:**

Considering reported death-censored graft survival in the SRTR dataset, we evaluated four models of molecular compatibility: antibody-verified Eplets, Snow, PIRCHE-II and amino acid matching. We have statistically evaluated their co-dependency and synergistic effects between models systematically on 400,935 kidney transplantations using Cox proportional hazards and XGBoost models.

**Results:**

Multivariable models of histocompatibility generally outperformed univariable predictors, with a combined model of HLA-A, -B, -DR matching, Snow and PIRCHE-II yielding highest AUC in XGBoost and lowest BIC in Cox models. Augmentation of a clinical prediction model of pre-transplant parameters by molecular compatibility metrics improved model performance particularly considering long-term outcomes.

**Discussion:**

Our study demonstrates that the use of multiple specialized molecular HLA matching predictors improves prediction performance, thereby improving risk classification and supporting informed decision-making in kidney transplantation.

## 1 Introduction

Recognition of donor tissue in transplantation poses a significant challenge for long-term survival of kidney grafts (1, 2). A major concern is the development of antibody-mediated rejection (ABMR), an immune-mediated process driven by HLA antibodies that target mismatched HLA proteins on the transplanted organ. This immune response can lead to graft dysfunction and, if left unaddressed, graft failure. ABMR has gained prominence as a leading cause of kidney graft loss, highlighting the significance of HLA antibodies in post-transplant complications (reviewed in (3)). In recent years, various approaches have emerged to assess the recipient’s probability of developing anti-donor HLA antibodies and achieving histocompatibility (4). These HLA antibodies, formed as a result of the recipient’s immune system recognizing foreign HLA antigens on transplanted tissue, can lead to a range of clinical problems after transplantation, including ABMR.

A detailed comprehension of how antibodies interact with mismatched HLA holds paramount importance in enhancing the matching between patients and donors, consequently improving clinical outcomes. Within this context, the identification of polymorphic amino acids situated on the surface of HLA proteins prone to incite an antibody response, has been manually delineated for a limited number of experimental crystal structures, giving rise to the concept of Eplets. This notion serves as the basic principle for the HLAMatchmaker algorithm (5). Subsequent advancements, such as EMS3D or HLA-EMMA, have further characterized HLA protein differences by considering physiochemical properties such as electrostatic dissimilarity or solvent accessible surface area (6–8). Eplet mismatch loads between patients and donors has been shown to demonstrate correlations with the formation of DSA (9, 10), graft rejection and organ loss (11). In addition, the use of EMS3D and HLA-EMMA models have also demonstrated utility in risk stratification for DSA and ABMR in the context of heart transplantation (12, 13).

The expanded availability of HLA crystal structures derived from a larger variety of alleles and improvements in the field of protein folding prediction opened opportunities to further fine-tune studies on accessibility of polymorphic amino acids on mismatched HLA by HLA-specific antibodies. The recently developed Snowflake algorithm used these new developments to analyze the surface area of mismatched amino acids while incorporating HLA protein-specific structural disparities (14, 15). The Snowflake concept was extended with the Snowball algorithm, which predicts local ellipsoid protrusion ranking to more robustly define antibody-accessible amino acid positions (together “Snow”) (16).

The development of donor HLA-specific antibodies is the result of a coordinated interplay between B cells and T cells, in which HLA-restricted antigen presentation by the B cell to a CD4-positive T-helper cell is essential (17, 18). The presented allo T-cell epitopes involved herein are derived from the B-cell targeted antigen, following the concept of linked recognition. To incorporate these concepts into risk stratification for HLA-specific antibody development, the PIRCHE algorithm was developed (19, 20). PIRCHE is a computational tool that quantifies the potential disparity between the HLA molecules of the donor and recipient at the level of T-cell epitopes. By quantifying these epitope mismatches, PIRCHE provides insights into the risk of developing HLA-specific antibodies after transplantation. Various retrospective studies have demonstrated associations between PIRCHE and transplant outcome (10, 21, 22). Consequently, PIRCHE aids in identifying patients who might be at a higher risk of immune responses against the transplanted organ due to HLA mismatches (23).

While HLA B- and T-cell epitope estimation algorithms have shown their individual predictive capacity, as outlined above, the concept of linked recognition suggests that an integrated approach enhances the precision of the risk classifications (13, 24–26). It is, however, not yet clear which biological factors play the most relevant roles in histocompatibility classification. We therefore evaluated the predictive performance of numerically combining different molecular compatibility algorithms accounting for the concept of linked recognition. To this end, we systematically analyzed Snow, amino acid mismatches, Eplets and PIRCHE for their impact on outcome by reanalyzing the publicly available dataset of 542,621 kidney transplants from the Scientific Registry of Transplant Recipients (SRTR). Moreover, we integrated and optimized the Snow and PIRCHE prediction algorithms and identified the most precise configurations to maximize risk stratification performance. Our data show that specialized B-cell and T-cell epitope predictions provide independent information on immunological risk classification, and that combinations of these predictors to supplement transplant characteristics provides further insights into histoincompatibility-driven risk.

## 2 Materials and methods

This study used data from the Scientific Registry of Transplant Recipients (SRTR). The SRTR dataset includes data on all donors, wait-listed candidates, and transplant recipients in the US, submitted by the members of the Organ Procurement and Transplantation Network (OPTN). The Health Resources and Services Administration (HRSA), U.S. Department of Health and Human Services provides oversight to the activities of the OPTN and SRTR contractors. The data reported here have been supplied by the Hennepin Healthcare Research Institute (HHRI) as the contractor for the SRTR. The interpretation and reporting of these data are the responsibility of the author(s) and in no way should be seen as an official policy of or interpretation by the SRTR or the U.S. Government. The study has been approved by the ethics committee of Virginia Commonwealth University (HM20030074).

**Figure 1:**
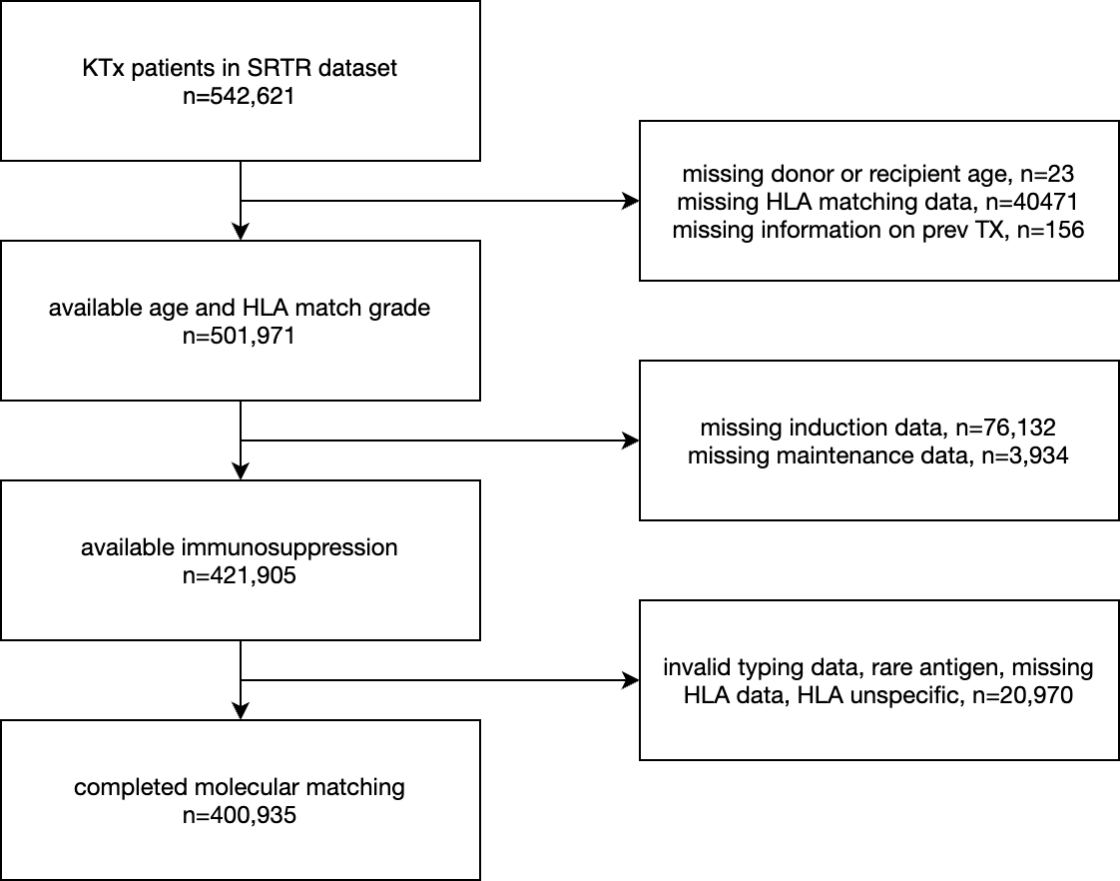
The subset of the SRTR kidney transplant dataset considered for statistical models.

The cohort considered a total of 542,621 kidney transplantation patients (data lock 2022-12-22). Low resolution HLA-A/-B/-DR typings of recipients and donors were used to apply molecular matching methods. Donor age, African American recipient, donor ethnicity, CMV mismatch, donor type and retransplantations were considered as known potential risk factors for model augmentation, forming a clinical reference model (27–32). Furthermore, recipient age (33) and Tacrolimus-based maintenance therapy (reviewed by (34)) were considered as known protective factors for model augmentation predicting death-censored graft survival. After data aggregation and filtering for missing data, a total of 400,935 cases were considered in statistical models (**Figure 1**).

Considering the overall SRTR dataset contains different epochs of kidney allocation, a subgroup analysis was performed for transplantations carried out between December 4th 2014 and December 3rd 2016 (i.e. two years), which reflects the implementation of a significant update to OPTN’s kidney allocation system (KAS). This subgroup consisted of 31,479 transplantations.

Four metrics of HLA molecular compatibility were considered within this study: Snow, amino acid mismatches, Eplets and PIRCHE-II, explained in the following.

### 2.1 Snow matching model

**Figure 2:**
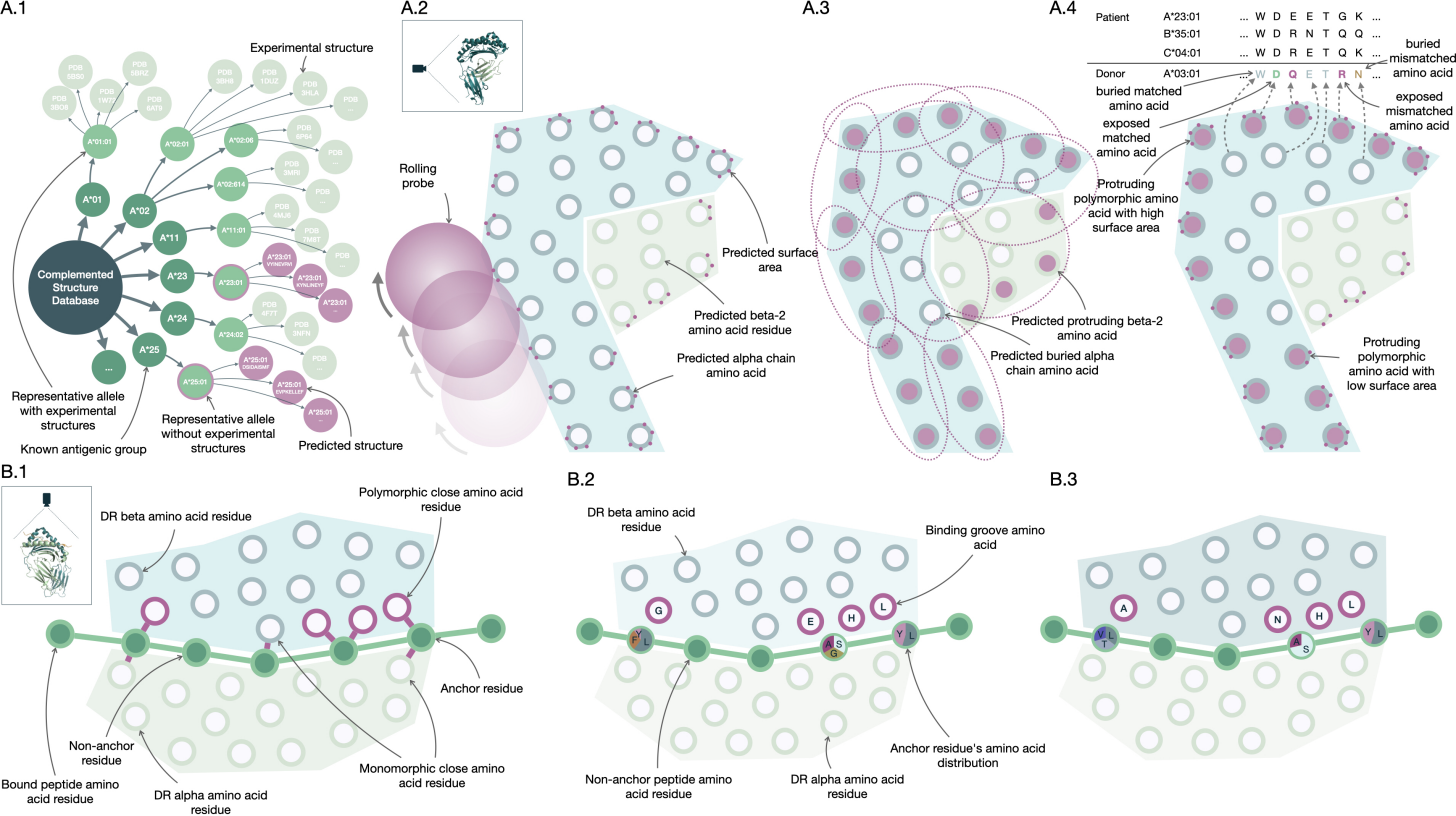
PIRCHE and Snow molecular compatibility algorithms. Construction of Snow **(A.1-4)** and PIRCHE-II **(B.1-3)** molecular matching algorithms. The Snow predictor fetches crystal structures of the Protein Data Bank (RCSB PDB, www.rcsb.org) and augments these with predicted protein structures **(A.1)**. For these HLA structures, Snowflake applies a rolling ball algorithm to predict surface area **(A.2)**, while Snowball uses repeatedly fitted ellipsoids to predict residue protrusion **(A.3)**. Neural networks trained on surface and protrusion data extrapolate the data to allow protein-specific antibody-accessible amino acid residue matching **(A.4)**. Based on HLA crystal structures, amino acid residues forming the binding HLA groove are identified for HLA-DR **(B.1)**. Experimental peptide HLA binding data curated in the Immune Epitope Database (IEDB, www.iedb.org) trains a neural network predictor **(B.2)**. Peptide and protein-specific binding affinity is inferred by a trained ensemble of neural networks to support prediction of donor-derived recipient HLA-bound peptides **(B.3)**.

The Snow algorithm (version 1.1) was utilized in this study to estimate the B-cell immunogenicity of mismatched HLA amino acids. It consists of two modules; Snowflake and Snowball. The Snowflake module evaluates the surface area of amino acids by a neural network that is trained on amino acid configurations and their respective surface area identified in publicly available crystal structures complemented by predicted HLA structures (**Figure 2A.1**). Amino acids exceeding a specified surface area threshold are considered exposed (**Figure 2A.2**). Exposed donor amino acids not present in the recipient’s self HLA proteins increases the Snowflake score by one. Notably, the Snowflake model takes the variability of surface area across HLA proteins into account, yielding protein-specific surface maps (14). For the present study, the Snowflake prediction pipeline was extended to additionally include the loci HLA-DRB1 and -DQB1 (16).

Considering solvent-accessible surface area alone without normalizing for respective amino acid size underestimates accessibility for small amino acids like glycine and overestimates it for large amino acids like tryptophan. To enhance the accuracy of prediction of antibody accessibility, the Snowball module has been suggested (16). Snowball employs a local ellipsoid protrusion ranking of amino acid positions. During the process, ellipsoids are iteratively fitted to substructures of atoms found in close proximity (within 15Å) to the current center atom (**Figure 2A.3**). The protrusion of atoms is subsequently ranked based on the ellipsoid’s axes, with the furthest atom receiving a rank of 1.0 and the atom centered in the ellipsoid receiving a rank of 0.0. By calculating the median of protrusion ranks for each structure’s atom and determining the maximum of atomic protrusion medians for each residue, the amino acid protrusion rank is defined. The Snow algorithm considers amino acid positions as exposed if they surpass both the Snowflake (surface area) and Snowball (protrusion) thresholds. Distinct, exposed donor amino acids mismatched with recipient self-HLA, increment the Snow score by one (**Figure 2A.4**, also named PIRCHE-B) (16). Mismatches derived from homozygous alleles are only counted once. The Snow matching algorithm is available via http://www.pirche.com for research purposes.

### 2.2 Amino acid matching

The sum of interlocus Class I and intralocus Class II amino acid configurations of donor HLA not present at the corresponding location in patient HLA was considered as the total number of amino acid mismatches. To that extent, the Snow algorithm was used without filtering amino acid mismatches by either surface area or protrusion.

### 2.3 Eplet matching

Eplet matching was carried out considering both all and antibody-verified Eplets listed in the HLA Eplet Registry (http://www.epregistry.com.br, accessed June 2022), respectively (35). Interlocus donor HLA-specific Eplets not present in the recipient’s self HLA-specific Eplets were considered as mismatched Eplets. The number of such Eplet mismatches is considered as Eplet mismatch score (6).

### 2.4 T-cell epitope matching

Donor HLA-derived T-cell epitopes presented by recipient HLA Class II were calculated by two versions of the PIRCHE-II prediction pipeline; the previously described PIRCHE-II version 3 and the newly released PIRCHE-II version 4.2 (36). PIRCHE version 3 (reviewed in (37)) counts the number of HLA-derived unique allo core peptides with a binding affinity below 1000nM as predicted by netMHCIIpan 3.2 (38), denoted as PIRCHE-II score (PIRCHE_II_v3). Following the previously suggested approach of normalizing PIRCHE-II scores by the respective presenting molecules’ predicted peptide binding promiscuity, a corrected PIRCHE-II score (PIRCHE_IIc_v3) was calculated (39).

PIRCHE version 4 was configured to use a newly developed peptide-HLA binding predictor named Frost for peptide binding by HLA-DRB1. In short, Frost predicts peptide binding using an optimum ensemble of 128 Artificial Neural Networks (ANN) selected from 512 randomly-initialized ANNs that had been trained and tested using binding data from the IEDB database csv export (https://www.iedb.org, accessed 2023/03/20) (40). These ANNs input BLOSUM-62 (41) encoded amino acids from a putative binding core, as well as encoded amino acids from the binding groove configuration of the presenting HLA-DRB1, considering two hidden layers of rectified linear units with Adam optimization (42). Twenty-five relevant binding groove positions were defined in HLA-DRB1 by identifying polymorphic amino acid residues in close proximity (< 4Å) with the bound peptide based on 22 HLA-DRB1 structures from the RCSB PDB (http://www.rcsb.org) (**Figure 2B.1**) (43). The ANNs are trained to minimize error from normalized and allele-specific binding affinities based on IC50 values (**Figure 2B.2**). The final ANN ensembles performed 1000 training iterations, which proved to be the best performing configuration.

Frost predicts a 9-mer binding core and an allele-specific binding affinity for every possible 15-mer peptide derived from HLA-A, -B, -C, -DRB1 and -DQB1. Subsequently, a binding rank is calculated by comparing the normalized affinity to predicted affinities of random 15-mers derived from human proteins considering the Uniprot protein database (44). The 128 ensemble ANNs vote on a predicted binding core, and a final binding rank is defined as the geometric mean of ranks from those networks which agree on the majority core. Model inference by the selected ensemble allows estimating binding affinity for proteins or peptides that haven’t been part of the training data (**Figure 2B.3**). The number of unique core peptide binders below a given permille rank threshold is considered as PIRCHE-II score (PIRCHE_II_v4, also named PIRCHE-T2). Peptides derived from homozygous donor loci are only counted once. As Frost incorporates binding affinity ranking, no binding promiscuity post processing is performed.

All PIRCHE-II analyses considered HLA-A, -B, -C, -DRB1 and -DQB1 presented by HLA-DRB1. PIRCHE versions 3 and 4.2 considered HLA protein sequences as reported by IPD-IMGT/HLA version 3.47 and 3.54, respectively (45). In case of missing exons, sequences were completed by an iterative nearest neighbor approach as previously described (46).

### 2.5 Imputation of protein-level typings

In order to convert the available low resolution HLA typing data into protein-level typings, the previously described multiple imputation approach was applied (47, 48). The reported race/ethnicity of an individual was mapped to either of the reported populations within the 2007 NMDP haplotype dataset (**Supplementary Table S1**) (49). HLA-C and -DQ alleles were imputed based on the available HLA-A, -B and -DR typing. The converted high-resolution haplotype pairs were filtered to a normalized threshold of 1%. For each high-resolution recipient-donor combination, molecular compatibility scores were calculated and aggregated by weighted summation of the respective pair’s frequency. This process has been automated and integrated as a web-service (http://www.pirche.com).

### 2.6 Statistical models

Death-censored graft survival was considered in two statistical survival models. Firstly, commonly used and well-understood Cox proportional hazard analyses were applied. Bayesian Information Criterion (BIC) was calculated on all models to identify optimal models. Lacking an external validation data set, Cox models fitted on 70% of the dataset (training data) were formed, using the remaining 30% of the data as test data for AUC calculation. The integral of AUCs (iAUC) were calculated considering the AUC at the 25th, 50th and 75th percentiles of follow-up time considering Uno’s suggested AUC estimator using the R package ‘survAUC’ (50, 51). To evaluate multicollinearity, variance inflation factors (VIF) were calculated for each models’ variables. Given that Cox models expect linear correlations but PIRCHE scores have been shown to be superlinear, log transformation of PIRCHE-II scores were performed as suggested previously by Lachmann et al. (10) To that extent, the uncorrected and corrected PIRCHE-II scores were incremented by one to prevent infinite values, followed by calculating the natural logarithm (PIRCHE_II_v3_log, PIRCHE_IIc_v3_log, PIRCHE_II_v4_log). Similarly, log-transformation was applied to Snow, amino acid and Eplet matching.

Secondly, the tree-based gradient boosting system XGBoost was applied as a representative of more recent learning algorithms (52). In particular, XGBoost was selected for delivering powerful predictions with acceptable computational efficiency, providing a freely accessible Python library and its comprehensive documentation. Accelerated failure time models with negative log likelihood models were trained on 70% of the dataset and evaluated on the remaining 30% of the data. Prediction performance was evaluated by iAUC and variable importance. Harrel’s Concordance Index was calculated for informational purposes only (53). Hyperparameters were systematically tested for number of trees (16 to 512, following the power of two) and maximum tree depth (2 to 12, with one increments), yielding 661 trained models per variable set. Early-stopping of training was allowed if model performance has not improved for a number of consecutive trees (0.5 times the maximum number of trees). A maximum number of 512 bins per feature was selected to compensate for the wide range of molecular matching scores. Variable importance was evaluated by gain metric. Log transformation was not applied, given the non-parametric nature of decision trees.

Model robustness was considered by ten repeats per model building, using different random seeds, which impacts both the initial split of training and test data, and following probabilistic decisions. BIC values of Cox models were scaled per random split to compensate for random seed-specific BIC magnitude. The corresponding median BIC was considered for ordering model performance. For XGBoost models, ensembles of the top 5 best-performing models considering iAUC of all tested hyperparameter configurations per random split were considered for competing model analyses.

Cox models were built and evaluated in R software (R 4.2.2, R Foundation for Statistical Computing, https://www.r-project.org). XGBoost models were built and evaluated in python (Python 3.10, Python Software Foundation, https://www.python.org/). Additional libraries have been listed in **Supplementary Table S2**.

### 2.7 Threshold optimization

Snow and PIRCHE-II configurations were systematically analyzed considering the Akaike Information Criterion (AIC), with Snowflake solvent accessibility and Snowball protrusion ranks ranging from 0.00 and 1.00. To limit computational runtime, Snow thresholds were incremented in 0.10 steps, with 0.02 increments between 0.20 and 0.70 (Snowflake) and 0.30 and 0.80. (Snowball), respectively. Threshold tuples of Snow were denoted as e.g. 0.22/0.54, which indicates a mismatched amino acid was considered as solvent accessible if the predicted Snowflake surface area score was above 0.22 and the Snowball protrusion rank was above 54%. PIRCHE ‰ ranks ranged from 5 ‰ to 500 ‰ with a superlinear distribution (PIRCHE_rank_ = [5, 10, 15, 20, 30, 40, 50, 75, 100, 150, 200, 250, 300, 400, 500]). Threshold triples of Snow and PIRCHE-II additionally denote the binding affinity rank of T-cell epitopes. In the threshold triple of e.g. 0.22/0.54/100, T-cell epitopes were considered as PIRCHE-II if their binding affinity rank was below 100‰ (i.e. 10%). Graft survival-specific optimal cutpoints for PIRCHE-II and Snow scores were estimated by maximally selected rank statistics as provided by the R survminer package.

## 3 Results

**Figure 3:**
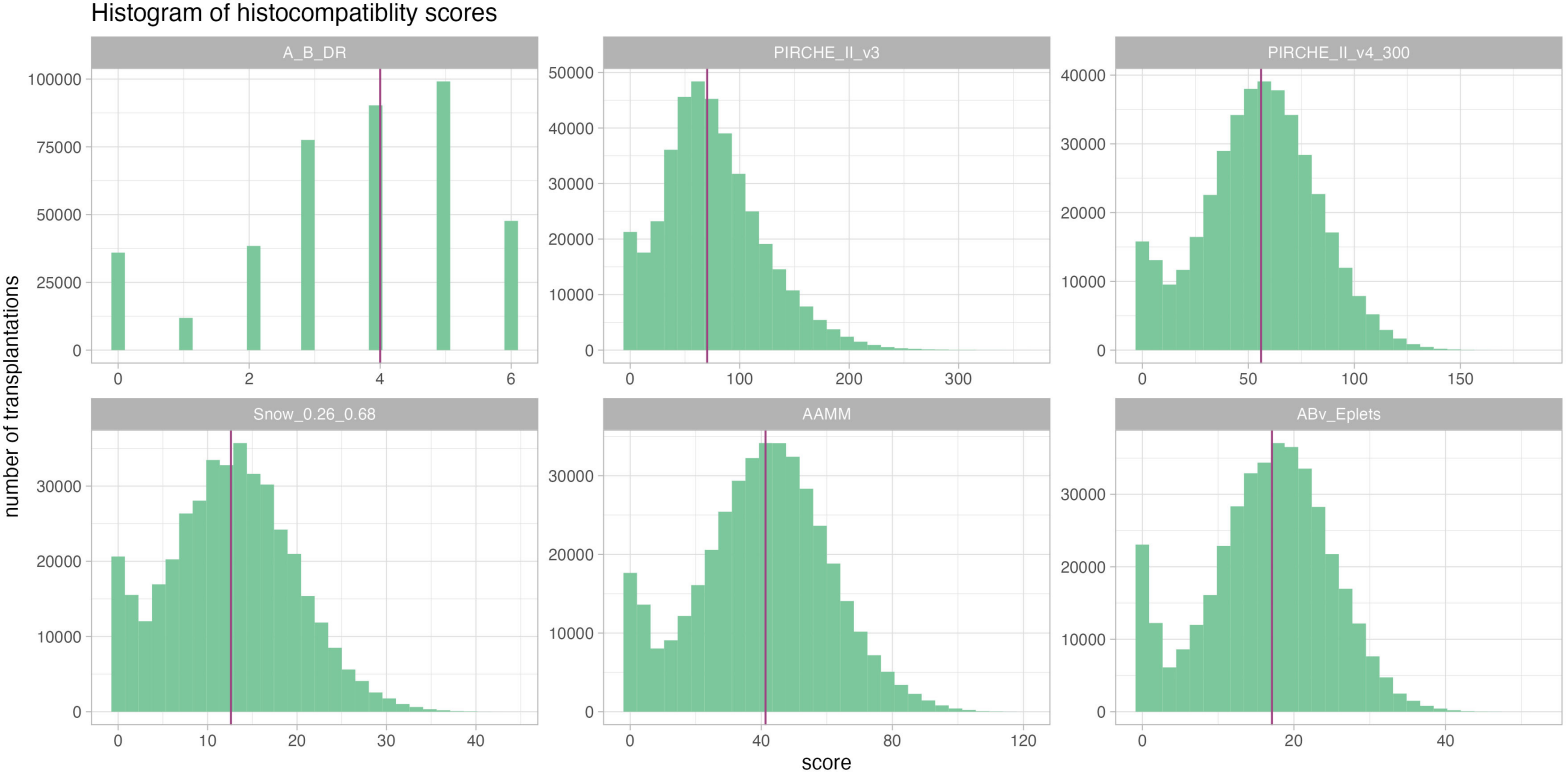
Histograms of the considered histocompatibility metrics reveal a comparable distribution pattern across all, albeit with very different numeric ranges. Median is given as a purple vertical line.

From the complete SRTR dataset, 400,935 kidney transplant cases were included in our analyses. Transplantations in this cohort were carried out between 1991 and 2022 (median 2010) and had a median follow-up time of 4.85 years (25th percentile: 1.98 years, 75th percentile: 8.97 years) with 76,077 reported graft losses (20%). Descriptive statistics of cohort demographics and distributions of molecular matching metrics are provided in **Table 1**; **Figure 3**.

**Table 1:** Demographic data of the 400,935 cases considered in the final histocompatibility analyses.

Variable	Percentage
Patient male	60.57%
Living donor transplantation	33.37%
Donor black	11.91%
Recipient black	24.44%
Tacrolimus maintenance	74.00%
CMV mismatch (Donor pos, Patient neg)	16.99%
First transplants	86.71%
Variable	Median (IQR)
Recipient age	51 (22)
Donor age	40 (23)
A-B-DR mismatches	4 (2)
PIRCHE-II v3	70.28 (58.06)
PIRCHE-II v4 (300‰)	56.00 (35.60)
Snow (0.26/0.68)	12.61 (9.52)
Amino acid mismatches	41.29 (26.51)
Antibody-verified Eplet mismatches	17.10 (10.59)
All Eplet mismatches	47.28 (25.76)

### 3.1 Threshold analysis for the SRTR cohort

Threshold analysis identified a Snowflake/Snowball configuration of 0.00/0.00 yielding optimal AIC (**Supplementary Figure S1**). Notably, this threshold configuration rendered the Snow algorithm identical to the number of donor amino acid mismatches. For PIRCHE version 4, the optimal AIC was reached at a comparatively weak binding affinity rank of 300‰, which is in line with previously reported weak optimal binding affinity cutoffs (10). When considering a multivariable model of both Snow and PIRCHE, more restrictive thresholds of the Snow model appeared beneficial, whilst the PIRCHE binding rank remained stable. The optimal AIC was reached at the Snowflake/Snowball/Frost threshold triple of 0.26/0.68/300 and improved over the individual models’ optimal AICs. Including HLA-A, -B and -DR serological matching to the multivariable model outperforms the previous model. The optimal threshold triple of 0.70/0.66/300 is only slightly better than 0.26/0.68/300, thus the latter configuration was considered as optimal Snow configuration in the proceeding analyses. Threshold analysis revealed the previously presented Snowflake algorithm (i.e. Snowball threshold fixed at 0.00) being outperformed by Snow in models combined with PIRCHE and HLA-A, -B and -DR matching. Performing three consecutive optimal cut point analyses per metric, optimal PIRCHE-II (version 4, 300‰) intervals are [0-23), [23-45), [45-69), [69-∞) (**Supplementary Figure S2**), optimal Snow (0.26/0.68) intervals are [0-17), [17-35), [35-47), [47-∞) (**Supplementary Figure S3**) and optimal antibody-verified Eplet intervals are [0-6), [6-16), [16-22), [22-∞) (**Supplementary Figure S4**). Supporting its validity, the optimal Snow configuration includes also the highly exposed amino acid positions of the majority of reported antibody verified Eplets (HLA-Class I, 50/74 = 67.6%; HLA-DRB1, 12/17 = 70.6%; HLA-DQB1, 21/31 = 67.7%; **Supplementary Figure S5**).

### 3.2 Competitive model analysis

**Figure 4:**
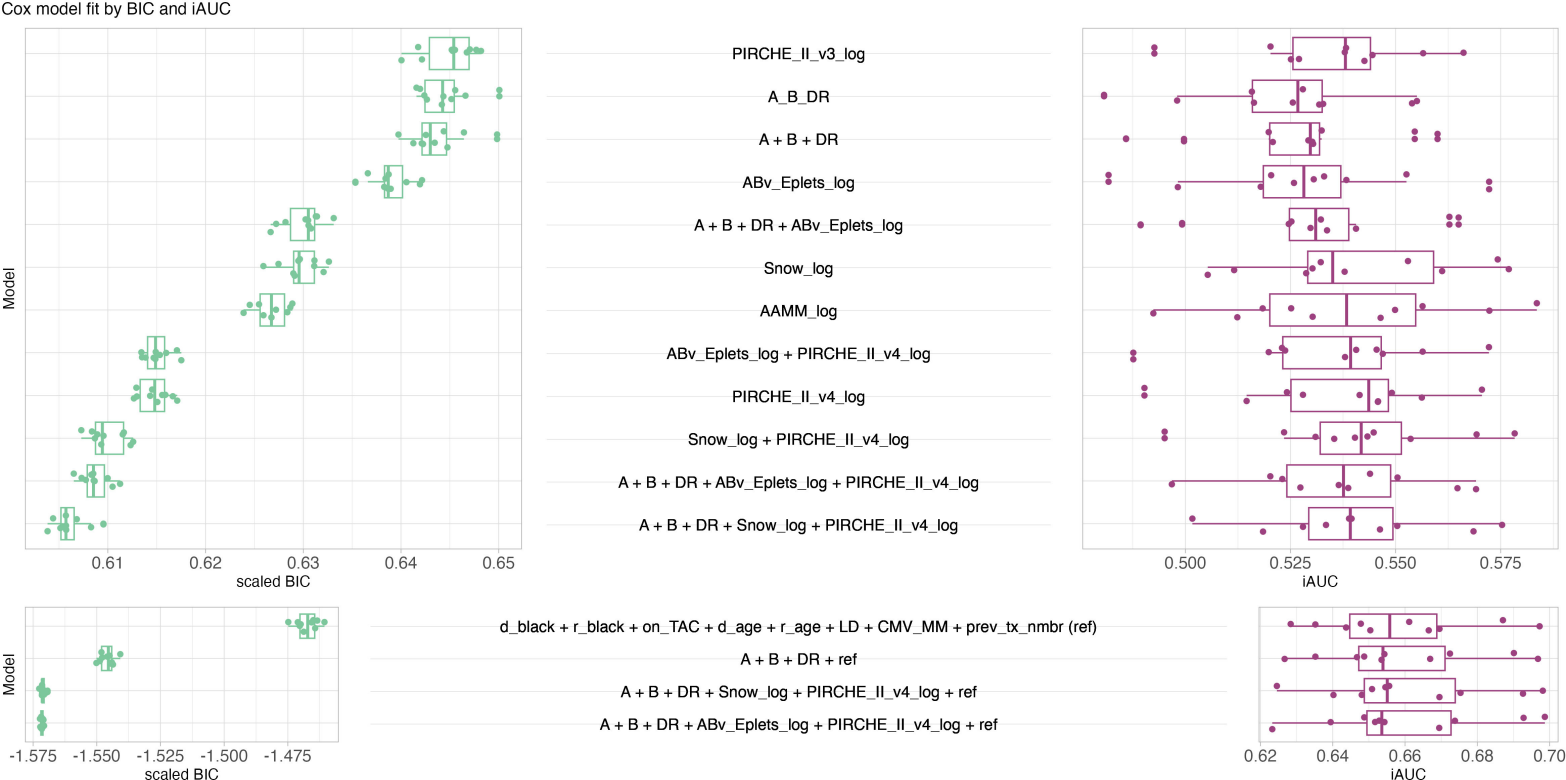
Butterfly plot of model performance of Cox models in terms of scaled BIC (green, left, lower is better, limitless numeric ordinal scale) and iAUC (purple, right, higher is better, range from zero to one). Each data point corresponds to a model fitted to 70% of the data and evaluated against the remaining 30% of the data. Top panel considers models only consisting of histocompatibility metrics, bottom panel considers models of known clinical risk factors in conjunction with histocompatibility metrics. The best histocompatibility model considers HLA-A, -B, -DR mismatching, Snow and PIRCHE-II. The best clinical models considered HLA-A, -B and -DR mismatch, PIRCHE and ABv Eplets or Snow, respectively. Boxplots depict the median (horizontal line), first to third quartile (box); the highest and lowest values within 1.5× IQR (whiskers) and outliers (circles), respectively.

The competitive model analysis revealed significant correlation of molecular matching scores and graft survival. Cox regression identified PIRCHE-II version 4 as the best univariable histocompatibility metric predicting graft survival (median iAUC = 0.5436), outperforming PIRCHE-II version 3, sum of A/B/DR mismatches (A_B_DR), Eplet, amino acid and Snow matching based on optimal BIC and iAUC (**Figure 4**). Molecular incompatibility loads appear superlinear with a dampened increase in hazard at high load scores, justifying log-transformation of these metrics (**Supplementary Figure S6**). Log-transformation improved BIC for all molecular matching metrics (**Supplementary Figures S7**, **S8**) with little to no impact on iAUC. Log-transformed antibody-verified Eplet load had a beneficial BIC over log-transformed all Eplet load. This effect persisted in multivariable models combined with PIRCHE (**Supplementary Figure S7**). Consequently proceeding analyses considered antibody-verified Eplets exclusively. All tested univariable models were statistically significantly correlated with graft survival (**Supplementary Figure S9**). The multivariable model of HLA-A, -B and -DR (A+B+DR) mismatches outperformed the univariable model of the summed number of mismatches on all three loci. Combining histocompatibility metrics in multivariable models generally outperformed models considering only their respective model components univariably. For example, Eplet matching combined with HLA-A, -B and -DR matching had a lower BIC and higher iAUC than either Eplet matching or HLA-A, -B and -DR matching alone. Considering only metrics of histocompatibility, competitive model analysis revealed the optimal BIC for a Cox model considering HLA-A, - and -DR matching, Snow and PIRCHE-II version 4 with a median iAUC of 0.5392. Considering Snow and Eplets in multivariable models simultaneously revealed elevated VIF for Eplet scores, suggesting their codependency (**Supplementary Figure S9**). Consequently, these covariate combinations were excluded from the analysis.

**Figure 5:**
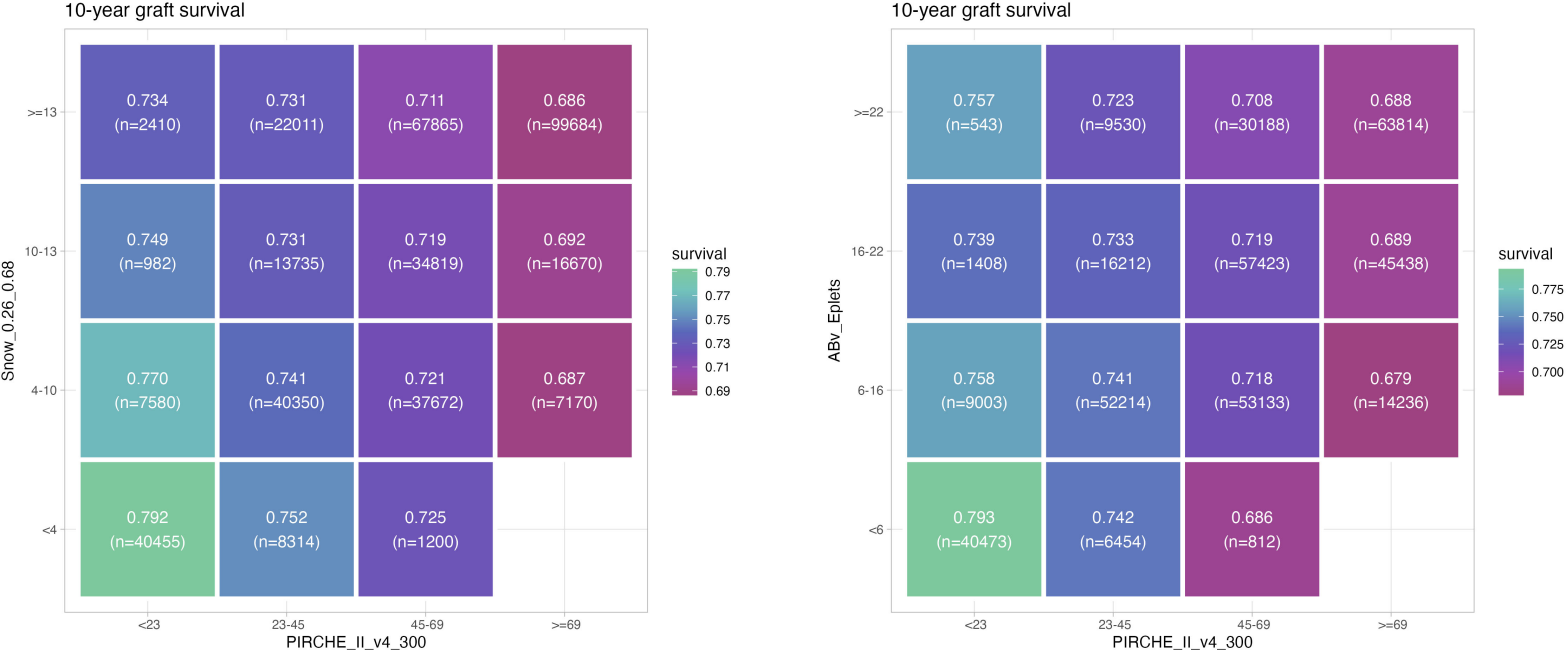
Estimated 10 year graft survival depending on PIRCHE-II category and Snow **(A)** or PIRCHE-II and Eplet category **(B)**, considering optimal cutpoints of the respective metrics (higher graft survival = green, lower graft survival = magenta). Numbers of patients per group are provided in braces. Example: Transplantations carried out with 6-16 Eplet mismatches have a 10 year graft survival ranging between 75.8% and 67.9%, depending on the PIRCHE-II being < 23 or ≥ 69.

Using the identified optimal cutpoints, estimated 10 year graft survival incidence was calculated for risk strata pairs of PIRCHE-II and Snow (**Figure 5A**), and PIRCHE-II and Eplets (**Figure 5B**), respectively. Notably within each risk group of a metric (i.e. within a column or within a row), a gradient in graft survival can be observed based on the respective second metric, indicating the additive value of the considered metrics for one another.

The clinical reference model (i.e. whether donor and patient are black, Tacrolimus maintenance immunosuppression, patient and donor age, living donor, CMV mismatch and the number of pre-transplants) outperformed histocompatibility models in predicting graft survival based on improved BIC and median iAUC of 0.6558. The addition of histocompatibility metrics further improved the model based on BIC, whilst iAUC remained similar. The optimal BIC was reached by augmenting the clinical reference model with HLA-A, -B, and -DR matching, Eplets and PIRCHE (**Figure 4**), with a very similar BIC in a model that exchanged Eplets by Snow. Considering locus-specific molecular matching scores showed only little impact on BIC. Additional model combinations were tested and its results provided in **Supplementary Figures S7**, **S8**.

**Figure 6:**
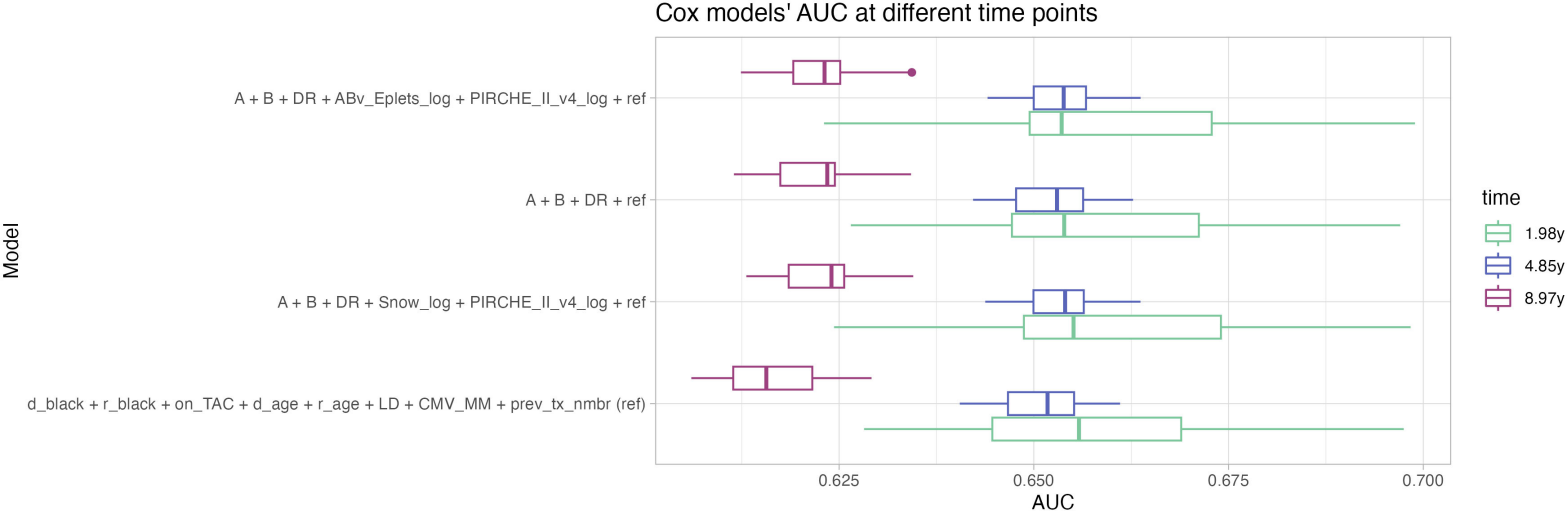
Distribution of repeated Cox models’ AUC at 1.98 years (green), 4.85 years (blue) and 8.97 years (magenta, corresponding to the 25th, 50th and 75th percentiles of the observation period) indicate higher performance of the histocompatibility-augmented models in long-term prediction. Boxplots depict the median (horizontal line), first to third quartile (box); the highest and lowest values within 1.5× IQR (whiskers) and outliers (circles), respectively.

Notably, the histocompatibility-augmented clinical models had a slightly worse short-term prediction performance at 1.98 years post transplantation. Opposed to that however, an increase in the long-term prediction performance could be observed for the clinical models that include histocompatibility and molecular matching metrics at 8.97 years post transplant (**Figure 6**).

**Figure 7:**
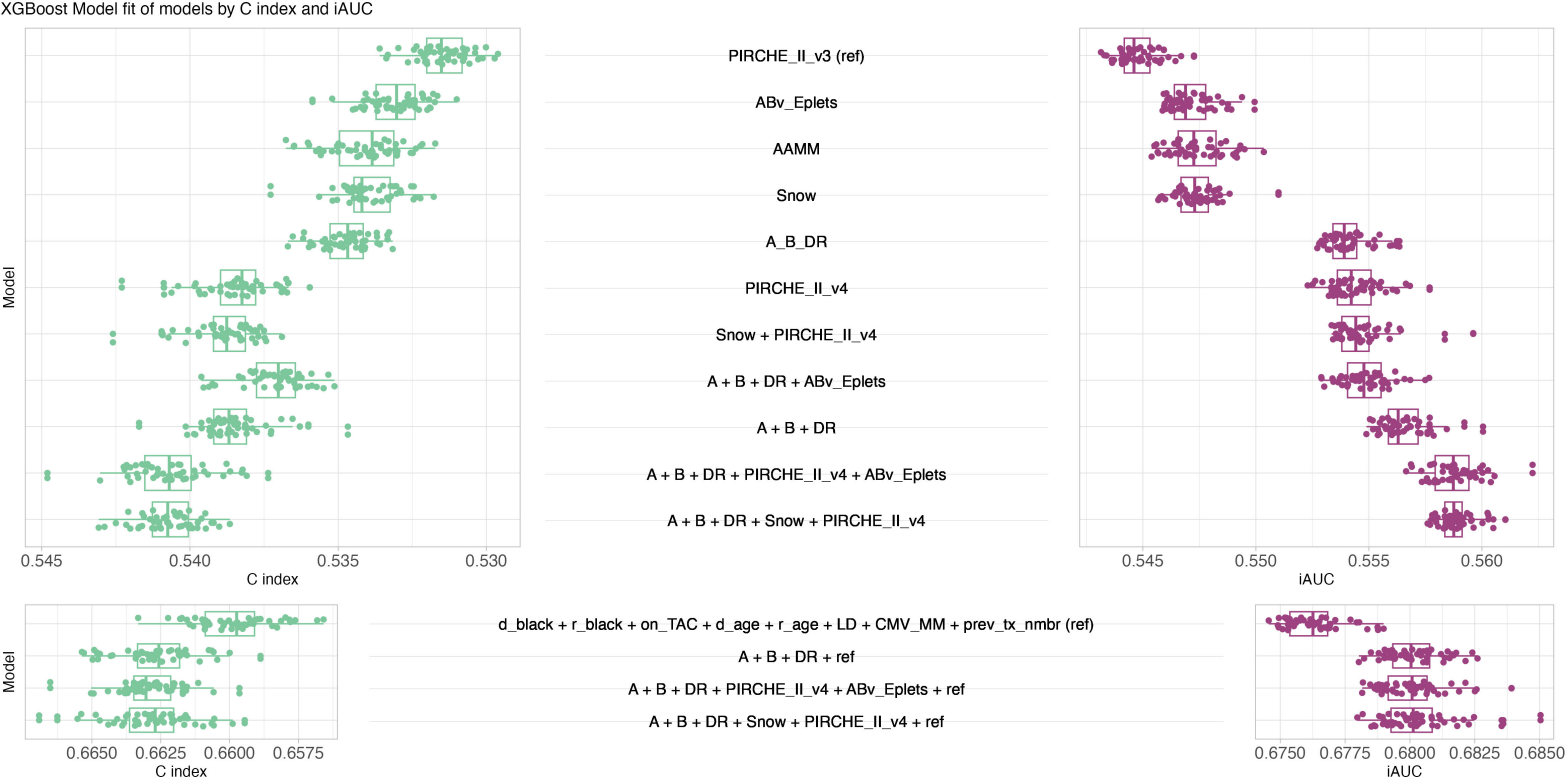
XGBoost model performance. Butterfly plot of fifty best XGBoost models’ performance in terms of C index (green, left, larger is better, ranging from zero to one) and iAUC (purple, right, larger is better, ranging from zero to one). Top panel considers models only consisting of histocompatibility metrics, bottom panel considers models of known clinical risk factors in conjunction with histocompatibility metrics. Boxplots depict the median (horizontal line), first to third quartile (box); the highest and lowest values within 1.5× IQR (whiskers) and outliers (circles), respectively.

XGBoost models were trained and hyperparameters were evaluated systematically considering C index (**Supplementary Figures S11**-**S13**) and iAUC (**Supplementary Figures S14**-**S16**). Optimal hyperparameter configurations per model and random split were selected for further analyses with median iAUC ranging between 0.545 for PIRCHE version 3 and 0.557 for an encompassing model of HLA-A, -B, -DR matching, Snow and PIRCHE. The model of known clinical risk factors improved further by joining it with HLA-A, -B, -DR matching, Eplets and PIRCHE, which resulted in C index and median iAUC of 0.680 (**Figure 7**). Similar performance was also observed when exchanging Eplets by Snow or ignoring molecular compatibility and relying solely on HLA-A, -B, -DR matching. Opposed to the Cox models, clinical XGBoost models augmented by histocompatibility metrics improved AUC time-independent (**Supplementary Figure S17**).

**Figure 8:**
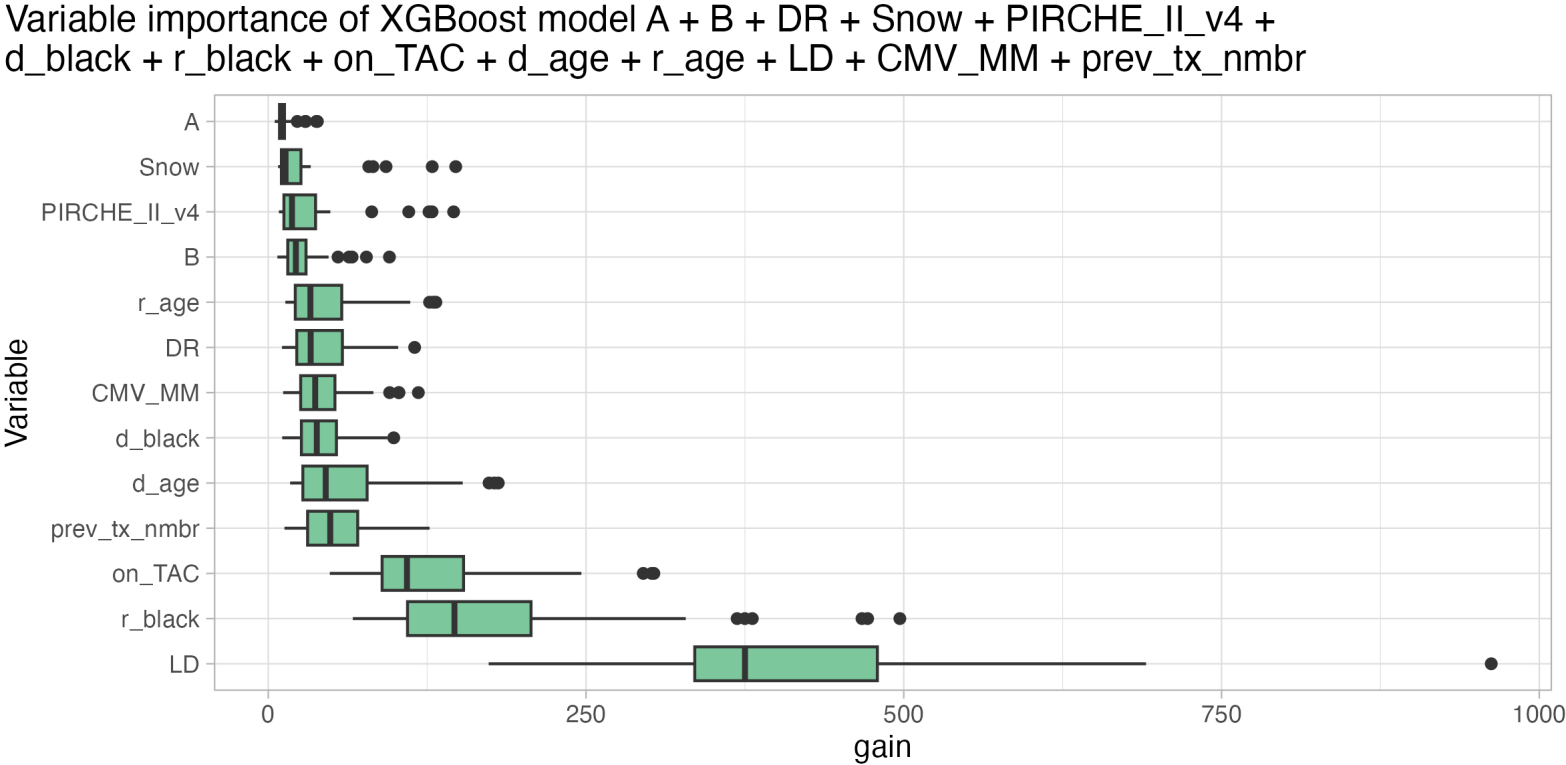
Variable importance based on gain metric (higher is better) of XGBoost model ensemble considering HLA-A (least important), -B, -DR matching, Snow, PIRCHE-II (PIRCHE_II_v4), donor being black (d_black), recipient being black (r_black), Tacrolimus maintenance (on_TAC), donor age (d_age), recipient age (r_age), living donor transplantation (LD, most important), CMV mismatch (CMV_MM) and number of previous transplantations (prev_tx_nmbr). Boxplots depict the median (horizontal line), first to third quartile (box); the highest and lowest values within 1.5× IQR (whiskers) and outliers (circles), respectively.

Variable importance of the best-performing XGBoost ensemble indicated that living donation was the most important variable, followed by recipient being black, Tacrolimus maintenance, donor age, number of previous transplantations, HLA-DR mismatch, recipient age, donor being black, CMV mismatch, PIRCHE, Snow, and HLA-B and -A mismatch (least important) (**Figure 8**). Notably, although HLA-A and Snow were least important in the model by an order of magnitude, their importance was still increased compared to an artificially added random variable, indicating their relevance (**Supplementary Figure S18**). The comparatively low importance of histocompatibility over other clinical parameters can to some extent be attributed to dividing histocompatibility into five individual input variables. In models that only consider one histocompatibility estimator, the variable importance of the respective metrics is still at a low level, but in the same order of magnitude as the recipient age (**Supplementary Figure S19**).

XGBoost models appeared to have a slightly better performance based on iAUC of up to 3%. This improved performance applied both for the histocompatibility-only models and the combined histocompatibility and clinical parameter models.

### 3.3 KAS 2014 subgroup analysis

The 2014 KAS subgroup considered 31,479 patients with a median follow-up time of 5.84 years (25th percentile: 4.28 years, 75th percentile: 6.16 years) with 3,496 reported graft losses (9%). Complex Cox models aggregating multiple concepts of histocompatibility mostly lack statistical significance (**Supplementary Figure S20**), although the individual metrics performed well in univariable analysis. Paired with clinical parameters a high scatter of iAUC can be observed (**Supplementary Figure S21**), suggesting underpowered models. Similarly, XGBoost models did not benefit from more complex histocompatibility models with HLA-A, -B, -DR matching being the strongest predictor of histocompatibility. Augmenting the clinical reference model with molecular matching did not improve them (**Supplementary Figure S22**). However, variable importance indicates that also XGBoost models suffer from insufficient power, with all parameters except from living donation and recipient being black not clearly exceeding the importance of a random control variable (**Supplementary Figure S23**).

### 3.4 Model inference

**Figure 9:**
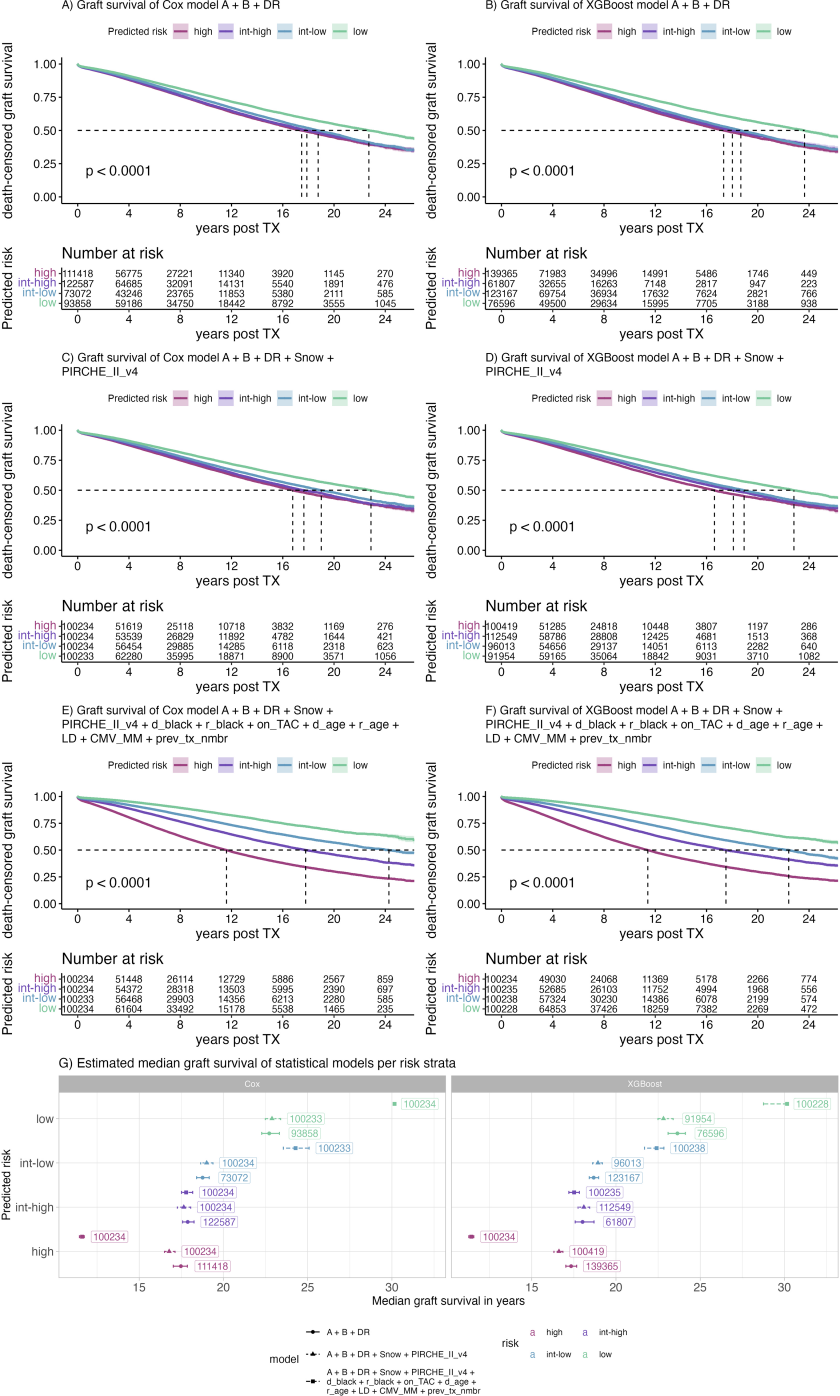
Kaplan Meier plots of selected Cox **(A, C, E)** and XGBoost **(B, D, F)** models predicting risk of early graft loss and their corresponding median graft survival **(G)**. Color indicates the risk predicted by the model ranging from low (purple) to high (green). Labels indicate the number of patients assigned to the respective risk category, panels indicate the respective model building framework, points indicate the median, error bars indicate the 95% confidence interval of the median graft survival.

Ensembles of Cox and XGBoost models were used to infer risk for all individuals. Patients of the full cohort were split into risk quartiles and Kaplan Meier plots of death-censored graft survival were generated (**Figure 9**). Given matching by HLA-A, -B and -DR is a coarse scale compared to the fine-grained molecular compatibility metrics, dividing patients considering the HLA-A, -B, and -DR matching model into risk quartiles did not yield equi-sized groups, with denser populated high-risk groups (**Figures 9A, B**). Conversely, models including Snow and PIRCHE matching yielded equi-sized groups with fewer patients being assigned the high-risk group and more patients being assigned the lower risk groups (low risk: delta = 6,375, intermediate-low risk: delta = 27,162) groups (**Figures 9C, D**). Despite these differences, Cox models including molecular matching distinguished graft half-life slightly better compared to HLA-A, -B, -DR matching (**Figure 9G**) (16.78y vs 17.47y, 17.65y vs 17.88y, 19.01y vs 18.76y, 22.88y vs 22.72y, for high, int-high, int-low and low risk, respectively). Although XGBoost models including molecular matching appeared to predict high-risk patients best, the advantage of the additional parameters appeared less pronounced (**Figures 9B, D**). Both in Cox and XGBoost models, the higher resolution scale of molecular matching appears to allow for better discrimination of patients in the lower and higher risk groups, reflected by the more even distribution of patients (**Figures 9A–F**).

## 4 Discussion

The immunological concept of linked recognition suggests the value of combining molecular compatibility models, as reported previously (24–26). The present study pioneers the application of simultaneous threshold analysis to optimally calibrate and combine molecular histocompatibility algorithms, offering a novel approach to aggregating specific pathways of HLA allorecognition for outcome prediction. Our findings underscore the importance of tuning these algorithms in conjunction. They suggested optimal configurations and thresholds for correlation with death-censored graft survival after kidney transplantation. Based on these optimal configurations, Cox and XGBoost models were trained on the continuous molecular matching metrics. A key strength of our study lies in its consideration of multiple molecular matching algorithms simultaneously within a large-scale kidney transplantation cohort. Moreover, with the substantial patient count in the SRTR dataset, we were empowered to conduct systematic threshold analyses.

Four distinct molecular compatibility algorithms were applied: Eplet matching considering antibody-verified Eplets (5, 54), amino acid matching restricted by protein-specific amino acid surface area and repeated localized protrusion rank (Snow) (16), prediction of indirectly recognizable HLA-derived T-cell epitopes (PIRCHE) (37), and the number of amino-acid mismatches, which also has been previously shown to associate with graft survival (55). Our results show that all four algorithms have a strong correlation with death-censored graft survival (**Figures 4**, **7**). This observation confirms previous reports of indirect T-cell epitope matching and Eplet matching in predicting graft survival in the SRTR dataset, respectively (56, 57). BICs, C statistics and iAUCs improved in multivariable histocompatibility models using both Cox and XGBoost models (**Figures 4**, **7**), suggesting additive value of the metrics. However, molecular mismatch load scores ignoring specific epitopes’ immunogenicity is a known limitation (58). Furthermore, the clinical impact of mismatches was shown to vary between HLA loci, with strongest correlations between incompatible HLA-DQ and immunological events (10). As such, future iterations of these algorithms considering immunogenicity-modeling are expected to improve performance.

Optimal configurations for Snow and PIRCHE in univariable and multivariable models were identified. The observed shift towards a more restrictive Snow configuration in multivariable models underscores amino-acid matching and to some extent classic antigen matching being good generalist models to predict impact of both the antibody pathway and indirect allorecognition pathway simultaneously, due to their inherent co-dependency on the HLA amino-acid sequences. A possible explanation could be that in simultaneous models, the individual predictors are allowed to specialize for the antibody pathway and indirect pathway, respectively. Considering donor-specific HLA antibody development as the observed variable, our presented optimal configurations of Snow and PIRCHE were applied and validated in an independent cohort of kidney transplant as reported by Chou-Wu et al (59).

Antibody epitope (i.e. Eplets or Snow) and indirect T-cell epitope models (i.e. PIRCHE) outperformed the predictive performance of amino-acid matching when applied in conjunction. This observation confirms the increased value of considering specialized prediction models for the different pathways of allorecognition. In Cox models combining Snow and Eplets, elevated variance inflation factors for Eplet and Snow scores confirm the conceptual and statistical similarity between the models (**Supplementary Figure S7**), which discourages their simultaneous use in the same statistical model. Consequently, combining either PIRCHE and Eplets or PIRCHE and Snow yields comparable prediction performance both in histocompatibility-only and combined clinical and histocompatibility models (**Figures 4**, **5**, **7**).

The analysis of known risk factors in kidney transplantation revealed additive value of combining molecular compatibility scores next to conventional HLA matching, both for Cox models and XGBoost models, confirming their importance for immunologic risk assessment. The performance of various model building frameworks to predict kidney allograft outcome has been reported to be in the same order of magnitude (60). However, a slight advantage of neural network-based models in predicting survival after liver transplantation has been reported (61). In our experiments XGBoost models appeared to have a better prediction performance with slightly higher iAUC than Cox models. This can likely be attributed to the non-parametric nature of tree-based learning algorithms, which maps the non-linear histocompatibility metrics better to immunologic risk. To account for overfitting, we’ve applied BIC over the Akaike Information Criterion, which penalizes excessive model sizes more heavily. Given the lack of a sufficiently powered external validation cohort in a similar population, we repeatedly trained and evaluated models considering randomly split data. We used Cox and XGBoost only as exemplary statistical frameworks for the purpose of evaluating relative improvements and interaction of molecular compatibility scores and did not aim to provide a comprehensive prediction model of clinical factors impacting post-transplant care decisions. Opposed to the iBox model (62), which primarily considers clinical parameters of the follow-up period, our models solely rely on pre-transplantation parameters, suggesting consideration of molecular matching in organ allocation and policy-making.

Although Cox models’ BIC improves when adding molecular compatibility scores to the reference prediction model of clinical parameters, the iAUC barely changes (**Figure 4**). However, evaluating the time-dependent AUC reveals that long-term prediction improves in models considering histocompatibility, which offsets the better short-term prediction of models absent of histocompatibility (**Figure 7**). The long-term advantage may also explain the limited impact of molecular matching seen in the 2014 KAS subgroup, given these patients had much shorter observed follow-up periods and fewer reported events. The domain-specific information contained in the HLA nomenclature and alleles’ serology continues being a major factor, confirming earlier results (63). However, with more covariates and non-parametric modeling, their additive value for prediction appears to decrease.

Our study’s retrospective nature and reliance on the SRTR dataset, which includes only low-resolution HLA typing information, may introduce biases or limitations in the findings. The imputation method for converting low-resolution HLA typing data to high-resolution typing, despite generally performing well (48), could introduce inaccuracies affecting the predictive performance of the models. Additionally, while our manuscript extensively validates the statistical performance of combining different histocompatibility models, it also underscores the complex multifactorial nature of predicting graft survival, which is clearly not exclusively affected by immunologic reasons. Although higher levels of histocompatibility appear beneficial for long-term graft survival, the models’ clinical utility may be stronger for predicting upstream events, such as antibody formation and graft pathology. Lastly, the practical implications of implementing these findings in clinical settings remain to be further explored, namely how these advanced molecular compatibility scores can be integrated into organ allocation systems, their potential in identifying patients for reduced intensity immunosuppression and their health economic value for personalized diagnostic monitoring schedules.

In summary, we demonstrated that combining the PIRCHE, and Snow or Eplet molecular compatibility algorithms with conventional HLA matching adds value in predicting kidney graft survival. We found optimal configurations being dependent on the combination of predictors and provided thresholds for univariable and multivariable models to best characterize contributions to outcomes. This suggests that combined specialized predictor models can improve antigen-level matching, and supports the hypothesis of linked recognition. It highlights the importance of simultaneously modeling and accounting for individual pathways of allorecognition. Further studies are needed to quantify the predictive performance of simultaneous histocompatibility models for immunologic events.

## Data Availability

The datasets presented in this article are not readily available because the Scientific Registry of Transplant Recipients manages access to its dataset. In a research context, PIRCHE and Snow scores can be calculated using the PIRCHE matching service at www.pirche.com. Requests to access the datasets should be directed to matthias.niemann@pirche.com.
