# Supplementary material for: Advancing risk stratification in kidney transplantation: integrating HLA-derived T-cell epitope and B-cell epitope matching algorithms for enhanced predictive accuracy of HLA compatibility
Source: Front Immunol. 2025 Feb 11;16:1548934. doi: 10.3389/fimmu.2025.1548934 (PMC11850546; doi:10.3389/fimmu.2025.1548934)
Supplement: Supplementary file 1 [file DataSheet1.pdf]

Supplementary Materials

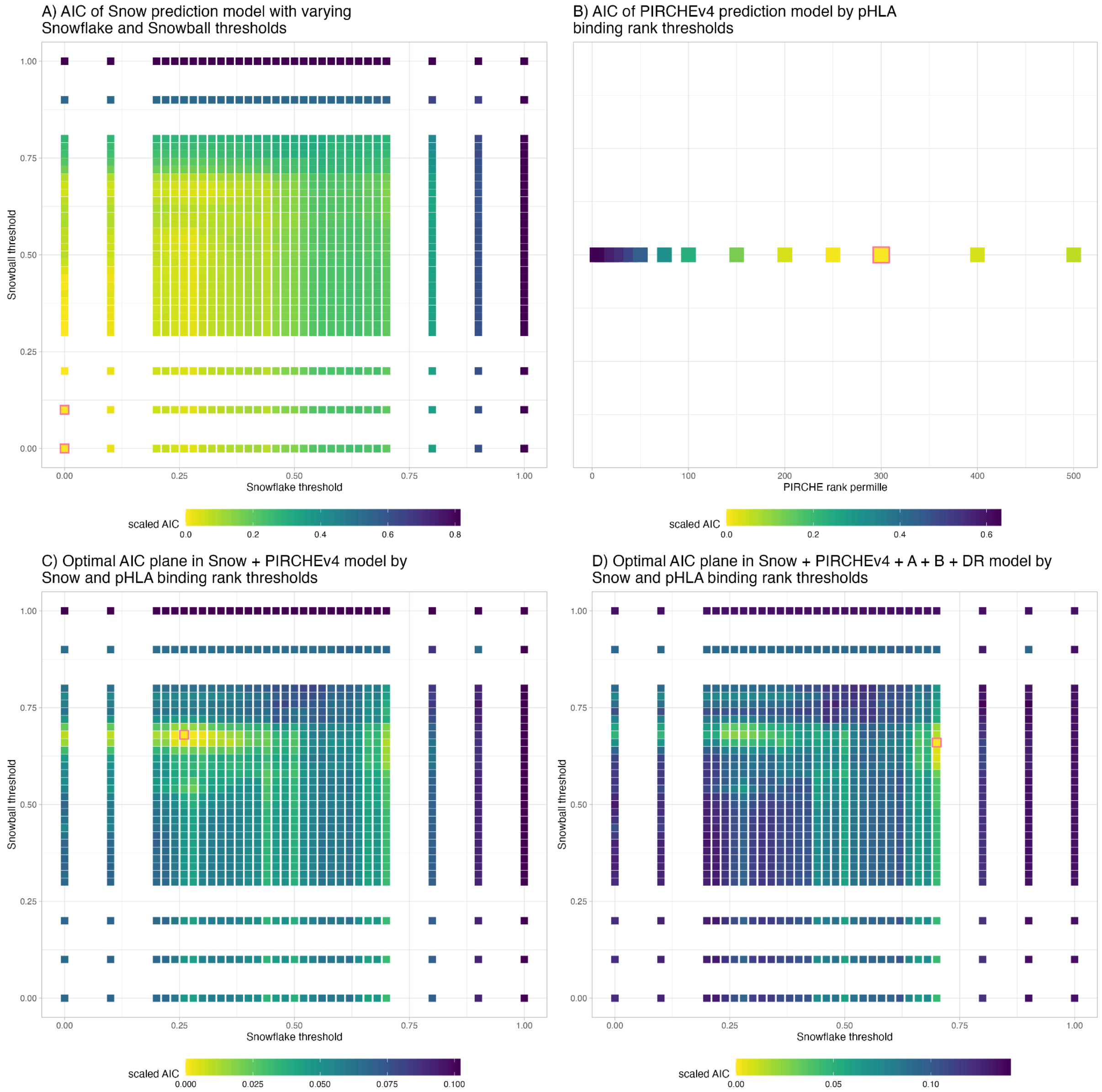

Supplementary Fig 1: Identifying thresholds for optimal AIC in Snow and PIRCHE models considering death-censored kidney graft survival in the SRTRC. Colors indicate the respective model's scaled AIC, with yellow indicating lower (better) values and purple indicating higher (worse) values. A) Optimal AIC (1911633) for a univariable Cox model considering only the log-transformed Snow score was reached at threshold pairs 0.00/0.00 (pink border). B) Optimal AIC (1911524) for a univariable Cox model considering only the log-transformed PIRCHE score was reached at a threshold of 300 ‰ (pink border). C) In a multivariable Cox model of Snow and PIRCHE, the optimal thresholds shifted towards a more restrictive 0.26/0.68/300 (pink border), yielding an AIC of 1911469. D) Adding HLA-A, -B and -DR matching to the multivariable Cox model shifted the optimal AIC (1911392) towards 0.70/0.66/300 (pink border). However, the configuration of 0.26/0.68/300 has a very similar performance with an AIC of 1911396. In a multivariable model, thresholds become more restrictive, possibly indicating a more specialized role for the individual predictors.

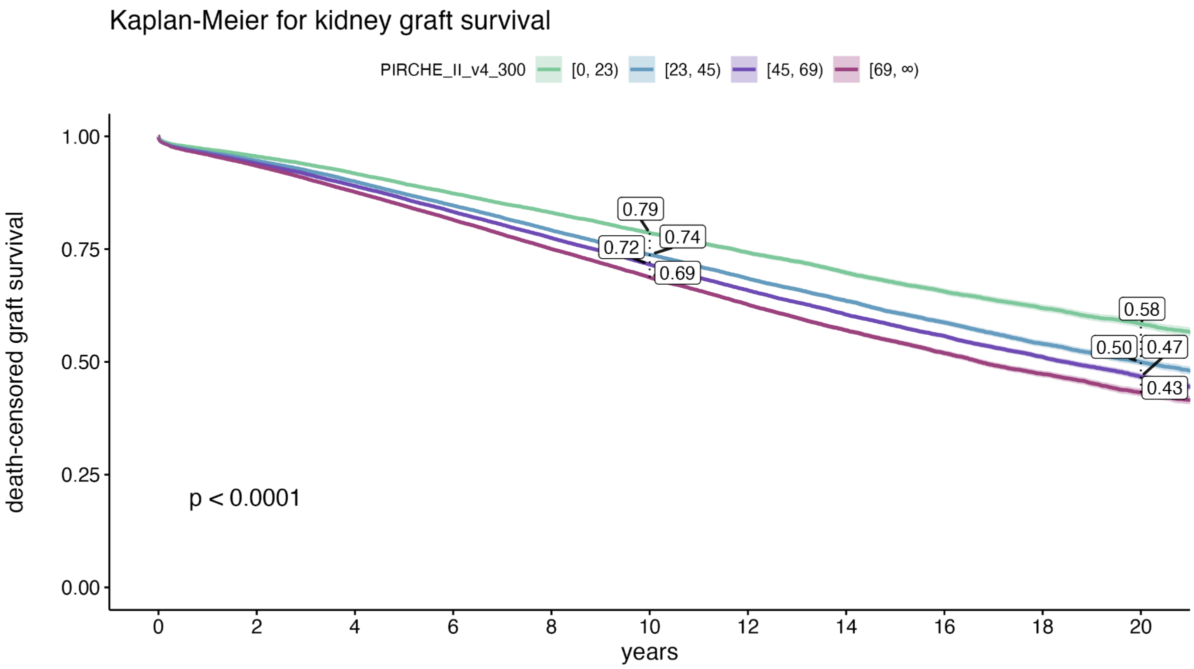

Number at risk

PIRCHE_II_v4_300	0	2	4	6	8	10	12	14	16	18	20
[0, 23)	51427	41428	33227	26019	20022	14928	10894	7691	5238	3479	2193
[23, 45)	84410	64444	49576	36931	27019	19395	13515	9161	6124	3938	2372
[45, 69)	141556	103897	77056	55789	39568	27099	18089	11715	7369	4452	2573
[69, ∞)	123542	88486	64033	45320	31218	20725	13268	8179	4901	2783	1564

years

Supplementary Fig 2: Kaplan-Meier plot considering optimal PIRCHE-II (version 4, 300‰) intervals (i.e. [0-23), [23-45), [45-69), [69-∞)) as identified by three consecutive cut point analyses using maximally selected rank statistics.

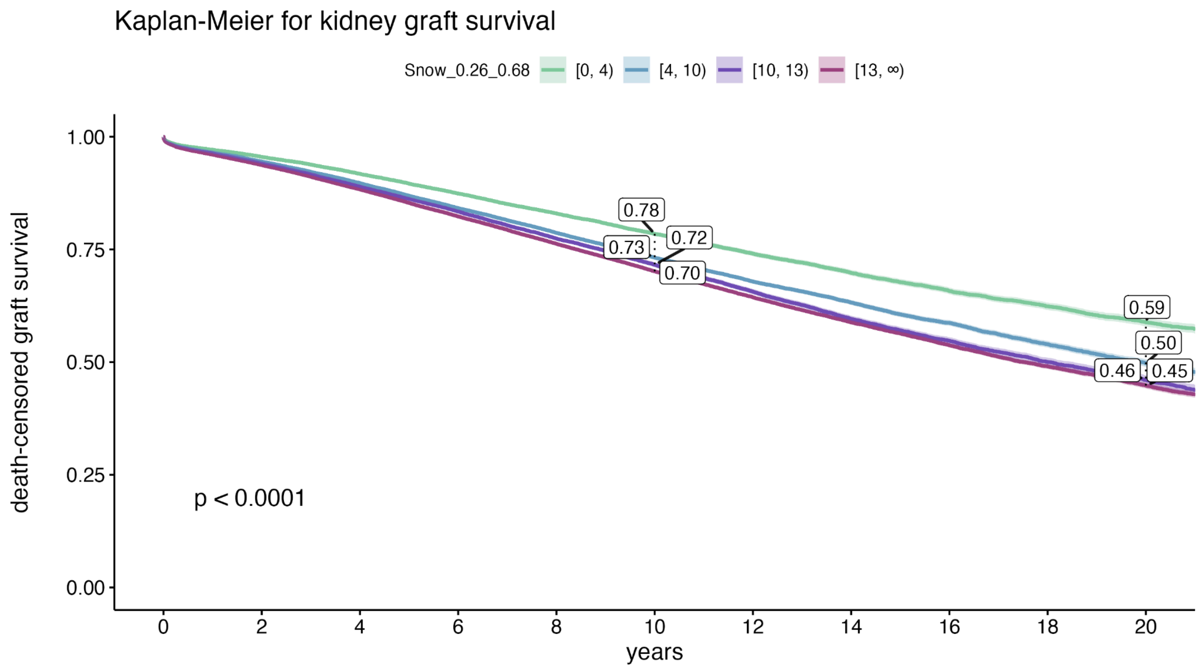

Number at risk

Snow_0.26_0.68	0	2	4	6	8	10	12	14	16	18	20
[0, 4)	49987	40118	32153	25144	19286	14405	10475	7437	5079	3393	2123
[4, 10)	92772	69953	52944	38945	28325	19970	13719	9217	6097	3860	2302
[10, 13)	66206	48761	36395	26525	18788	13027	8695	5580	3510	2124	1281
[13, ∞)	191970	139423	102400	73445	51428	34745	22877	14512	8946	5275	2996

years

Supplementary Fig 3: Kaplan-Meier plot considering optimal Snow (0.26 Snowflake surface area threshold, 0.68 Snowball protrusion rank threshold) intervals (i.e. [0-4), [4-10), [10-13), [13-∞)) as identified by three consecutive cut point analyses using maximally selected rank statistics.

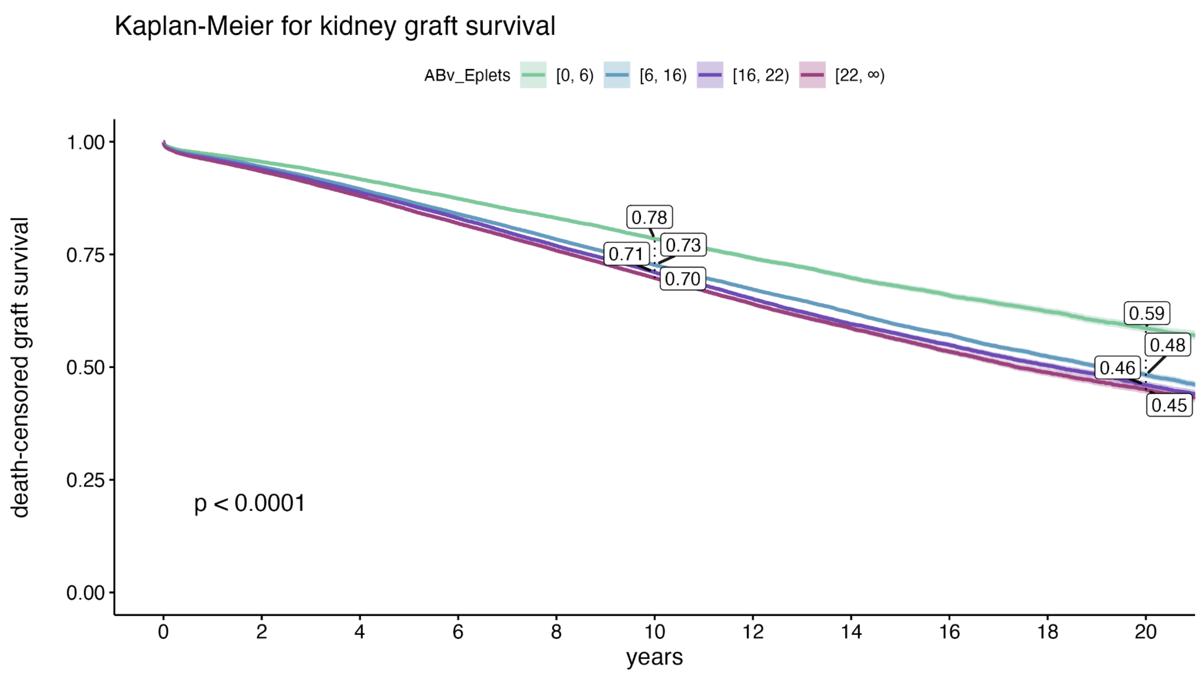

Number at risk

ABv_Eplets	0	2	4	6	8	10	12	14	16	18	20
[0, 6)	47793	38618	30980	24369	18806	14053	10294	7260	4944	3296	2069
[6, 16)	128586	96900	73624	54245	39299	27775	19047	12762	8453	5331	3202
[16, 22)	120481	87946	64842	46768	32940	22394	14795	9472	5919	3581	2068
[22, ∞)	104075	74791	54446	38677	26782	17925	11630	7252	4316	2444	1363

years

Supplementary Fig 4: Kaplan-Meier plot considering optimal Eplet intervals (i.e. [0-6), [6-16), [16-22), [22-∞)) as identified by three consecutive cut point analyses using maximally selected rank statistics.

Solvent accessibility vs. protrusion rank of most exposed positions of reported antibody-verified Eplets

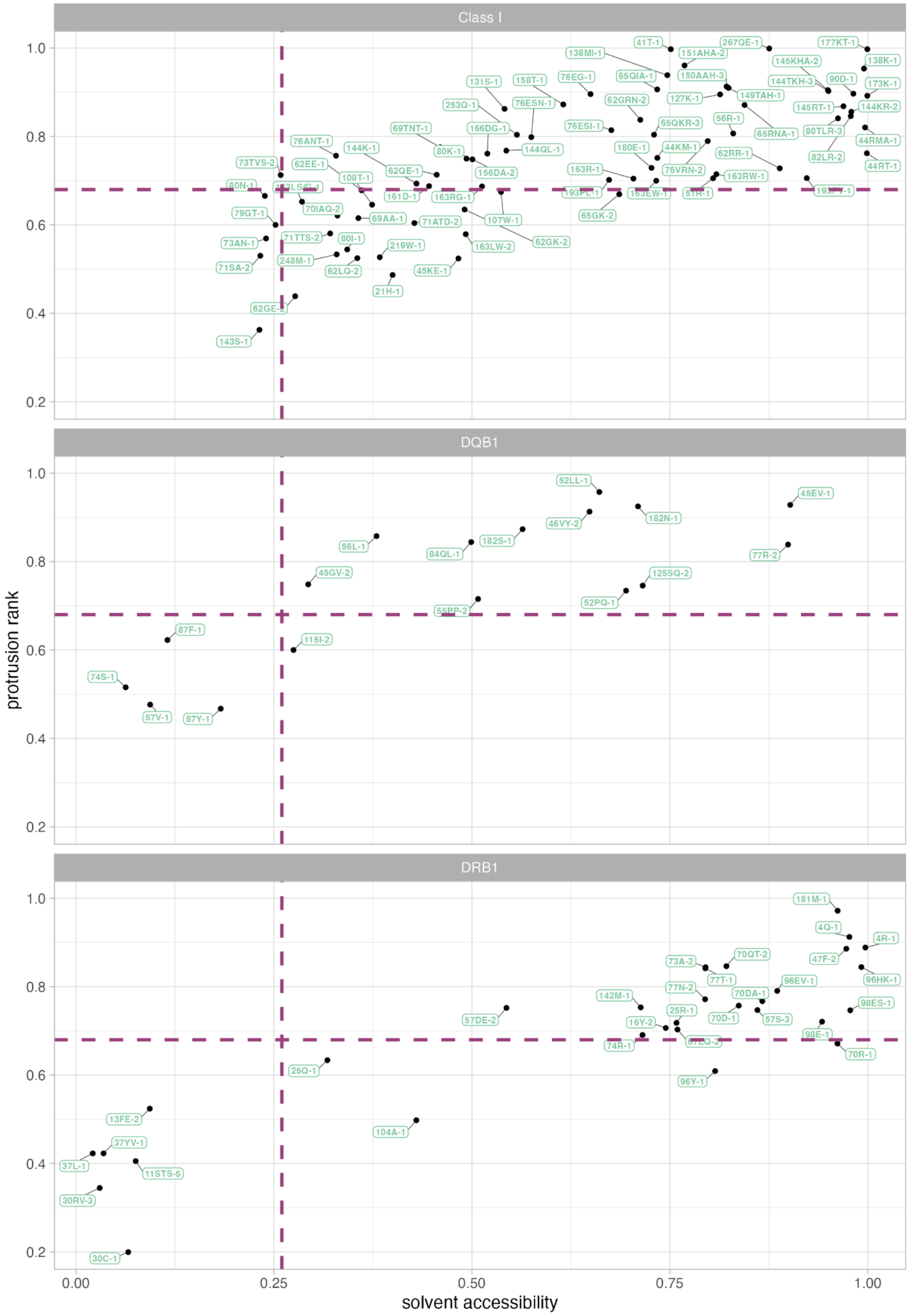

Supplementary Fig 5: The identified optimal accessibility by surface area (x axis) and protrusion rank (y axis) includes the majority of antibody-verified Eplets' exposed positions.

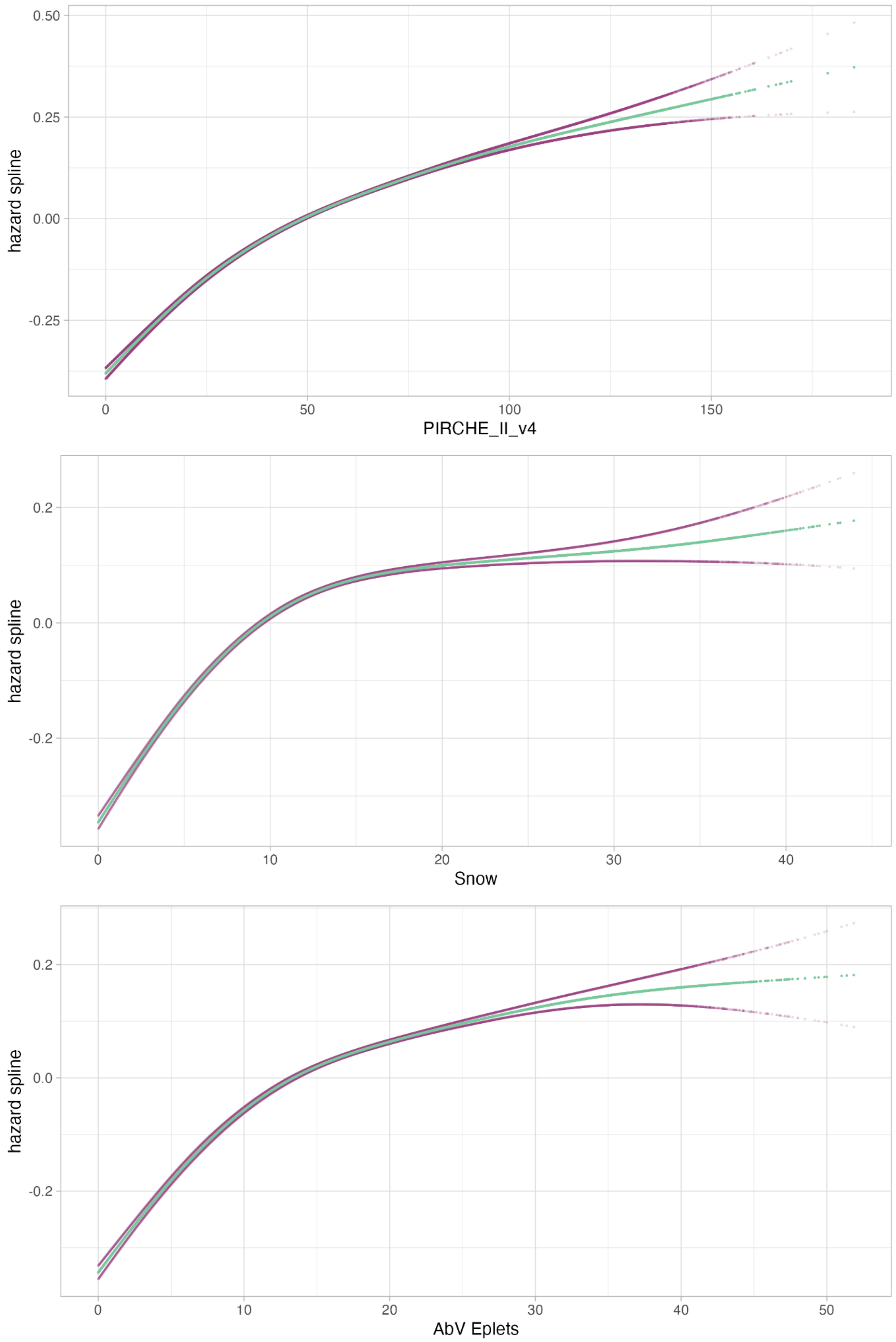

Supplementary Figure 6: Spline of coefficient in Cox regression considering PIRCHE, Snow and Eplets indicate the need for transformation prior to application in linear models.

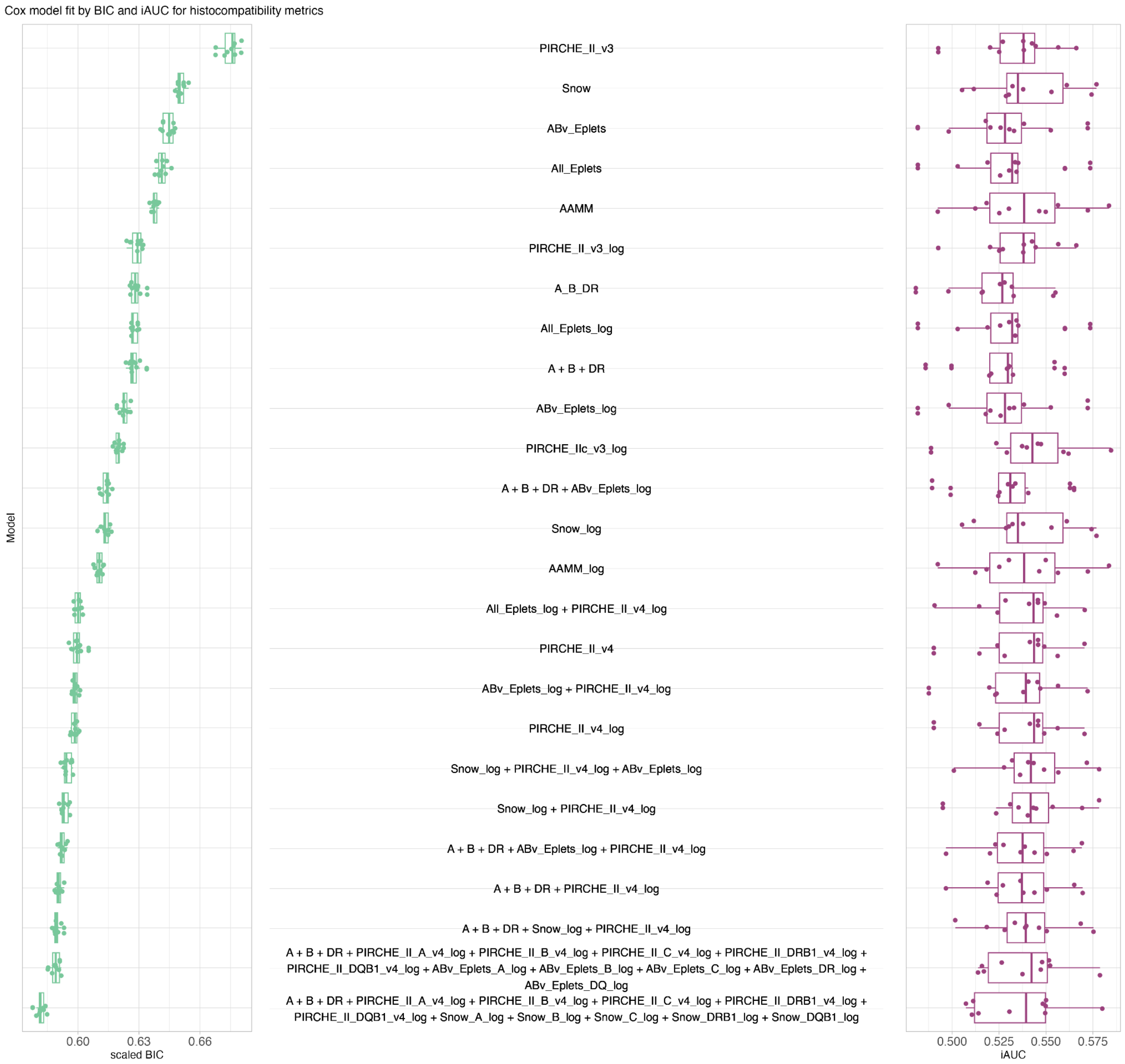

Supplementary Fig 7: Extended butterfly plot of scaled BIC (green, left, lower is better) and mAUC (purple, right, higher is better) of Cox proportional hazards models considering histocompatibility metrics predicting graft survival using the overall cohort. Boxplots depict the median (horizontal line), first to third quartile (box); the highest and lowest values within 1.5× IQR (whiskers) and outliers (circles), respectively.

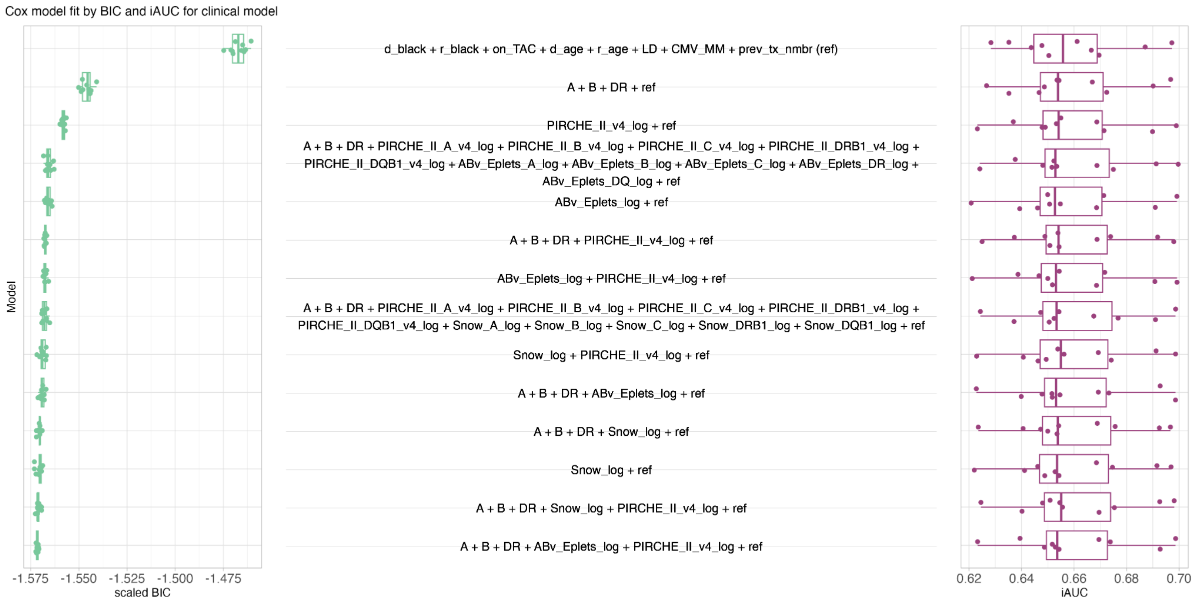

Supplementary Fig 8: Extended butterfly plot of scaled BIC (green, left, lower is better) and mAUC (purple, right, higher is better) of Cox proportional hazards models considering augmented clinical parameters predicting graft survival using the overall cohort. Boxplots depict the median (horizontal line), first to third quartile (box); the highest and lowest values within 1.5× IQR (whiskers) and outliers (circles), respectively.

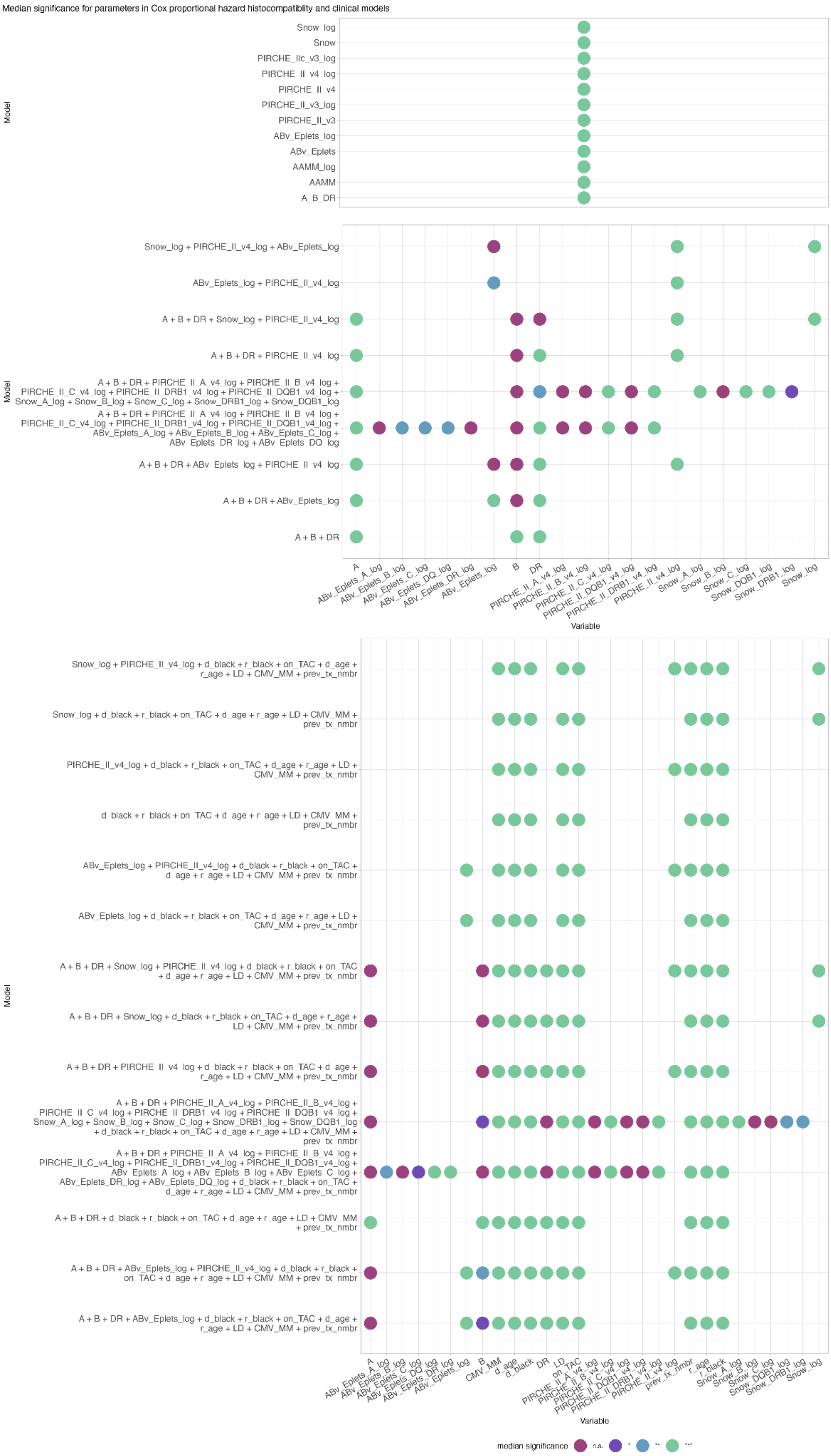

Supplementary Fig 9: Median significance levels of repeated Cox proportional hazard models considering the full cohort. Certain models lack significance for some of its variables, e.g. HLA-A matching in the models considering the clinical parameters and molecular matching simultaneously. n.s.: p >= 0.5, *: p < 0.05, **: p < 0.01, ***: p < 0.00

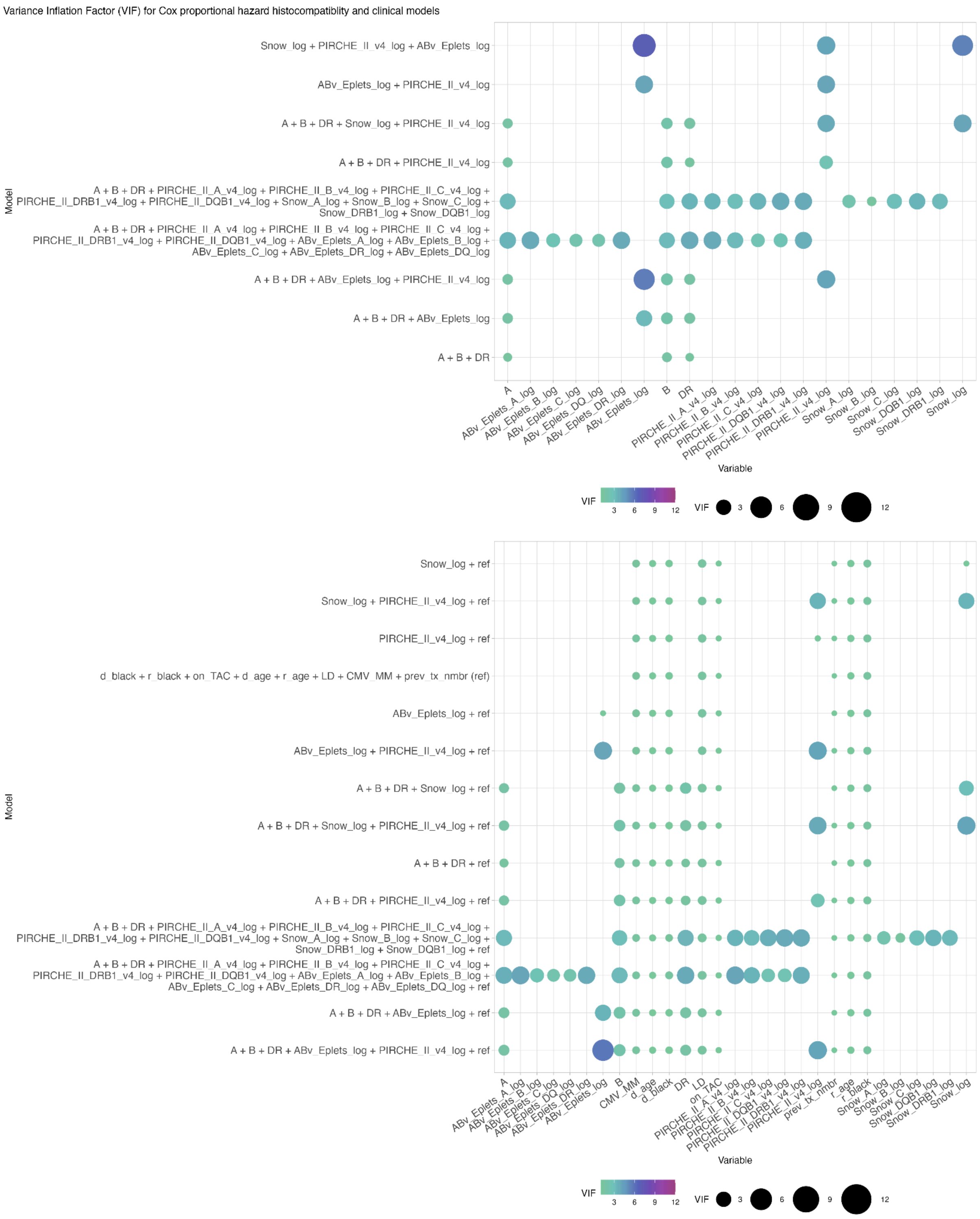

Supplementary Fig 10: Cox variance inflation factors (VIF) for multivariable prediction models. In particular for models containing both Eplets and Snow, elevated VIF can be observed, suggesting these variables are codependent.

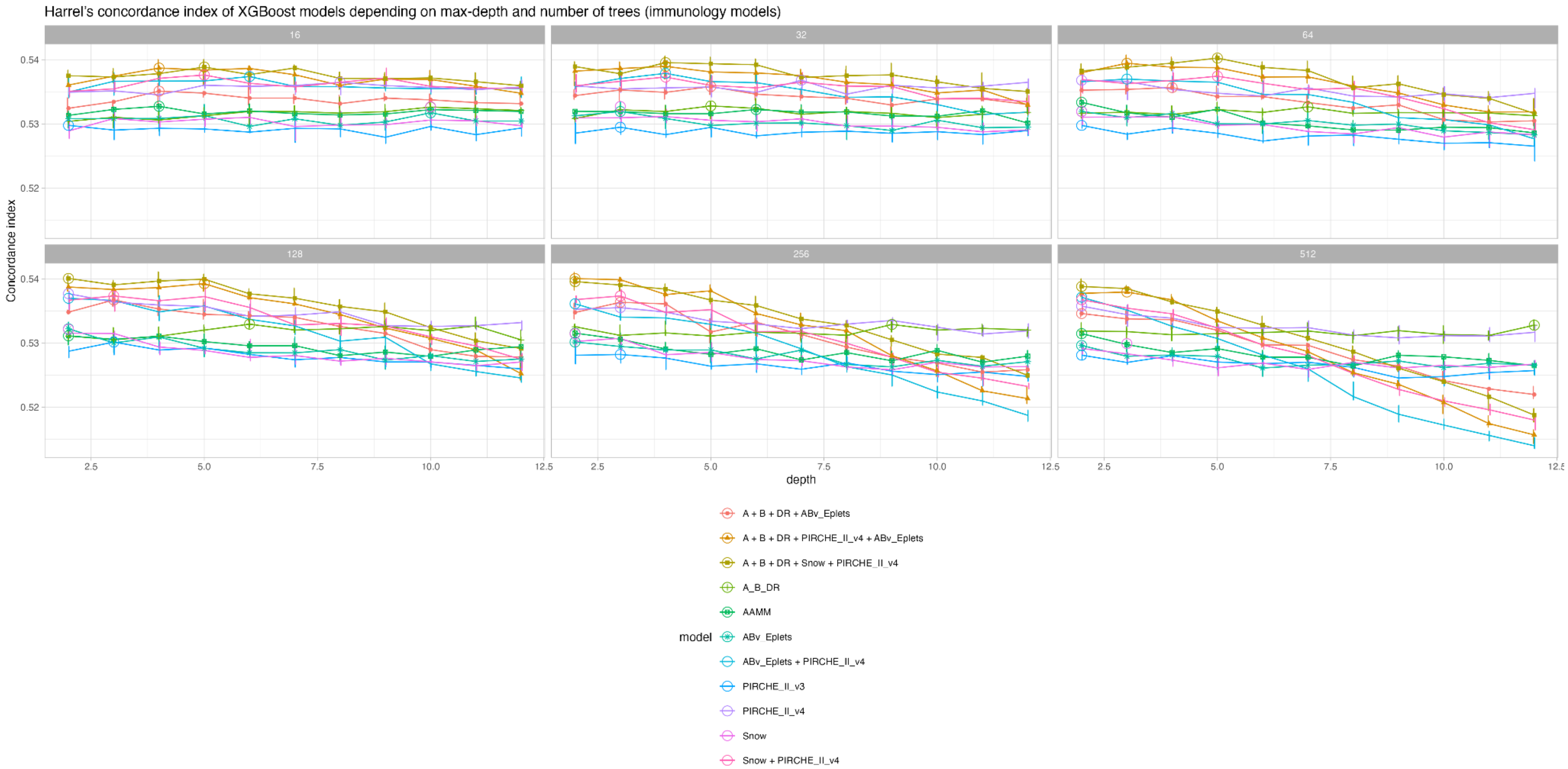

Supplementary Fig 11: Concordance index (y axis) of repeated XGBoost models of molecular matching sum scores considering maximum model depth (x axis) and number of trees per model (panels). Points indicate the respective median C index with the error bars indicating the first and third quartiles. Circles around points indicate the respective models maximum median value per panel.

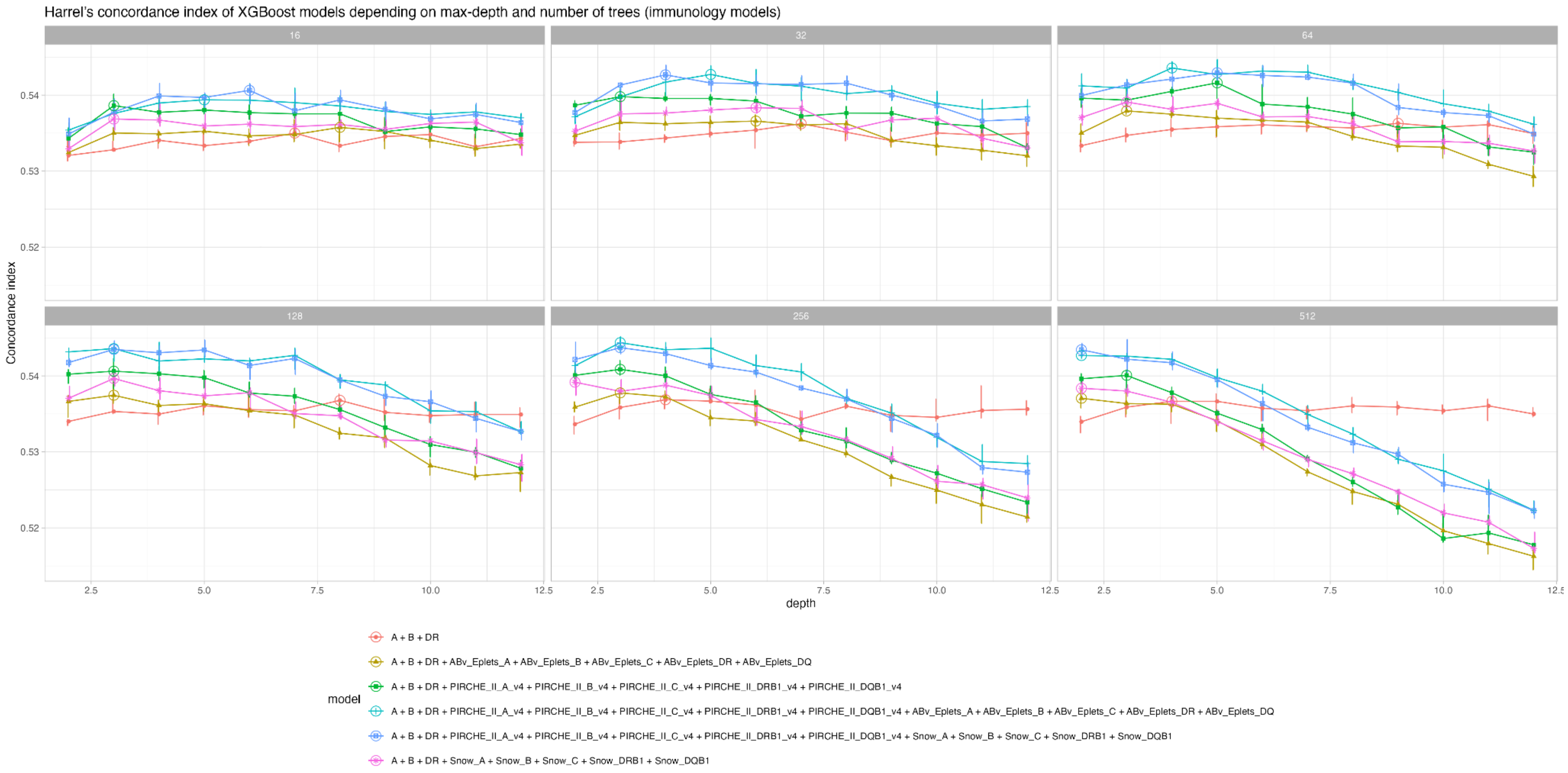

Supplementary Fig 12: Concordance index (y axis) of repeated XGBoost models of molecular matching scores per locus considering maximum model depth (x axis) and number of trees per model (panels). Points indicate the respective median C index with the error bars indicating the first and third quartiles. Circles around points indicate the respective models maximum median value per panel.

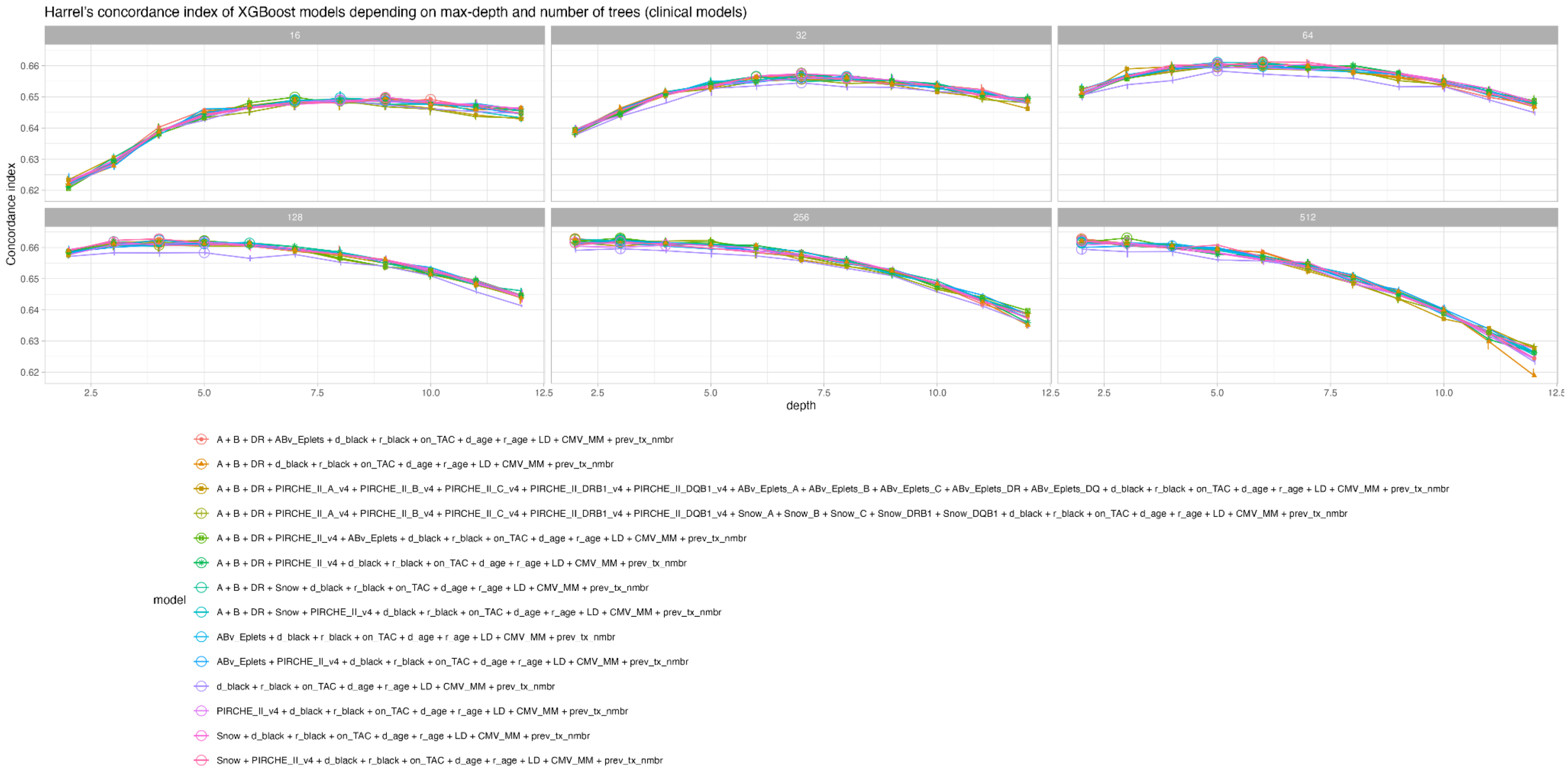

Supplementary Fig 13: Concordance index (y axis) of repeated XGBoost models of clinical parameters considering maximum model depth (x axis) and number of trees per model (panels). Points indicate the respective median C index with the error bars indicating the first and third quartiles. Circles around points indicate the respective models maximum median value per panel.

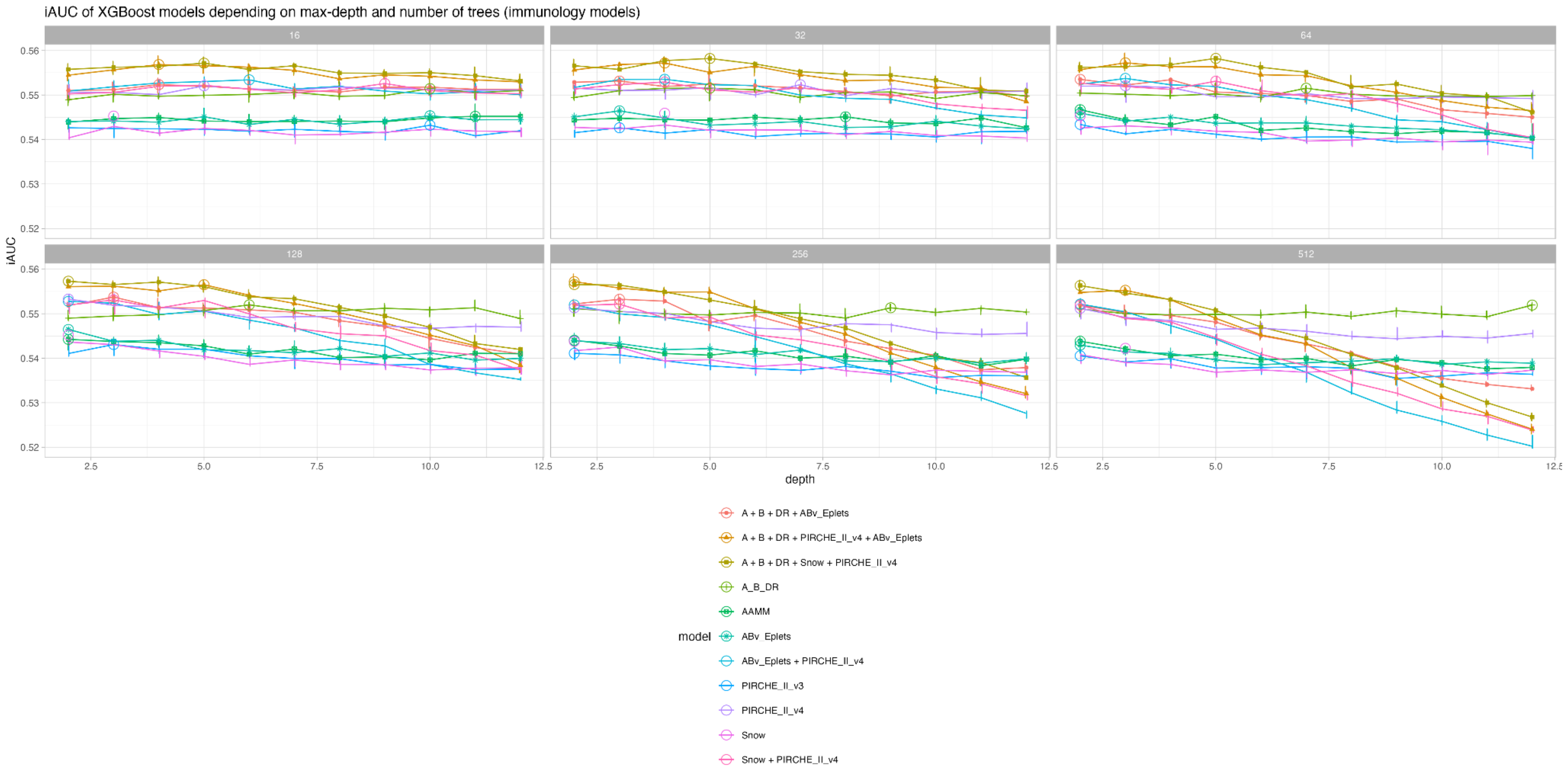

Supplementary Fig 14: Integrated AUC (y axis) of repeated XGBoost models of molecular matching sum scores considering maximum model depth (x axis) and number of trees per model (panels). Points indicate the respective median C index with the error bars indicating the first and third quartiles. Circles around points indicate the respective models maximum value per panel.

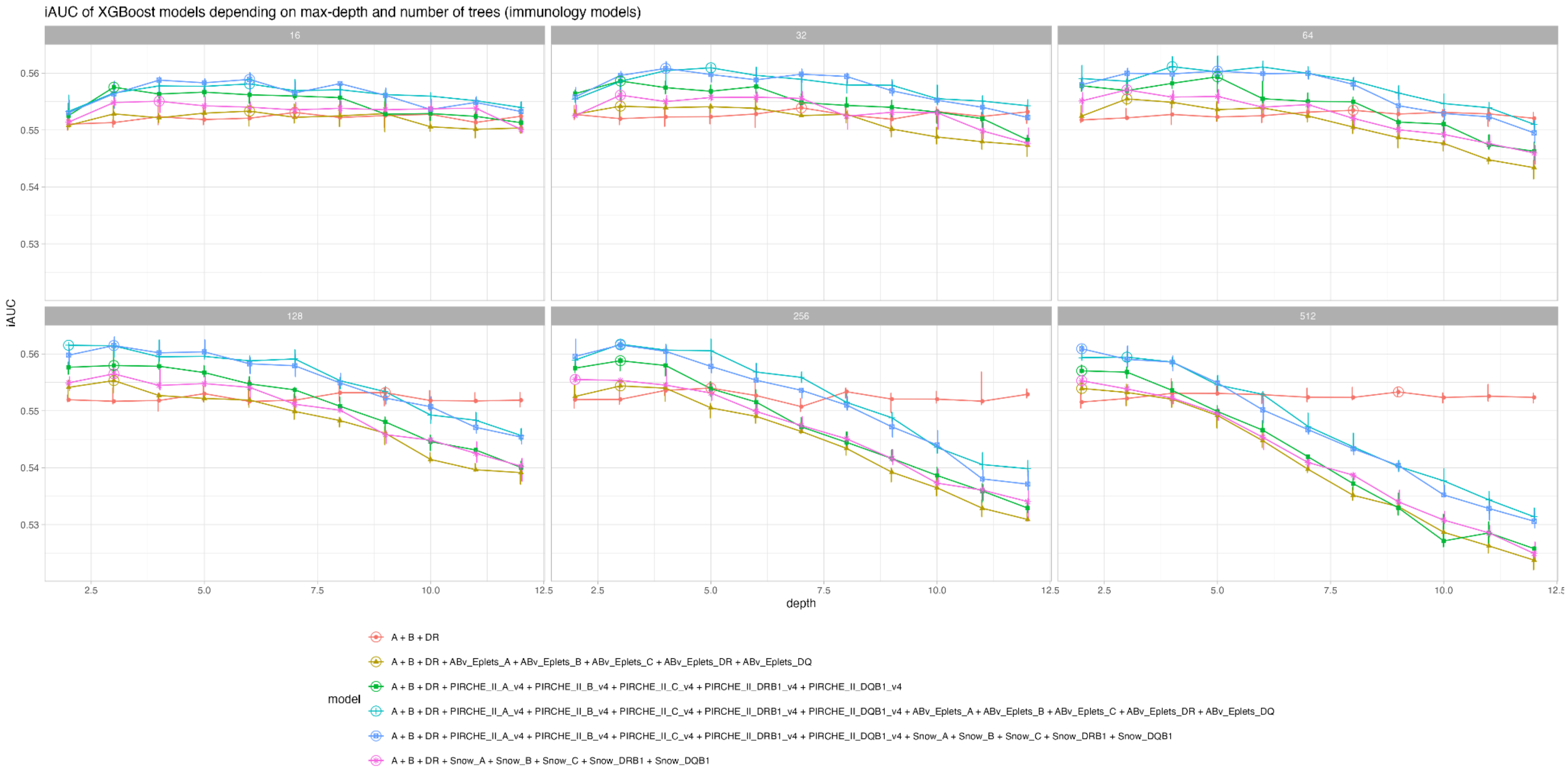

Supplementary Fig 15: Integrated AUC (y axis) of repeated XGBoost models of molecular matching scores per locus considering maximum model depth (x axis) and number of trees per model (panels). Points indicate the respective median C index with the error bars indicating the first and third quartiles. Circles around points indicate the respective models maximum median value per panel.

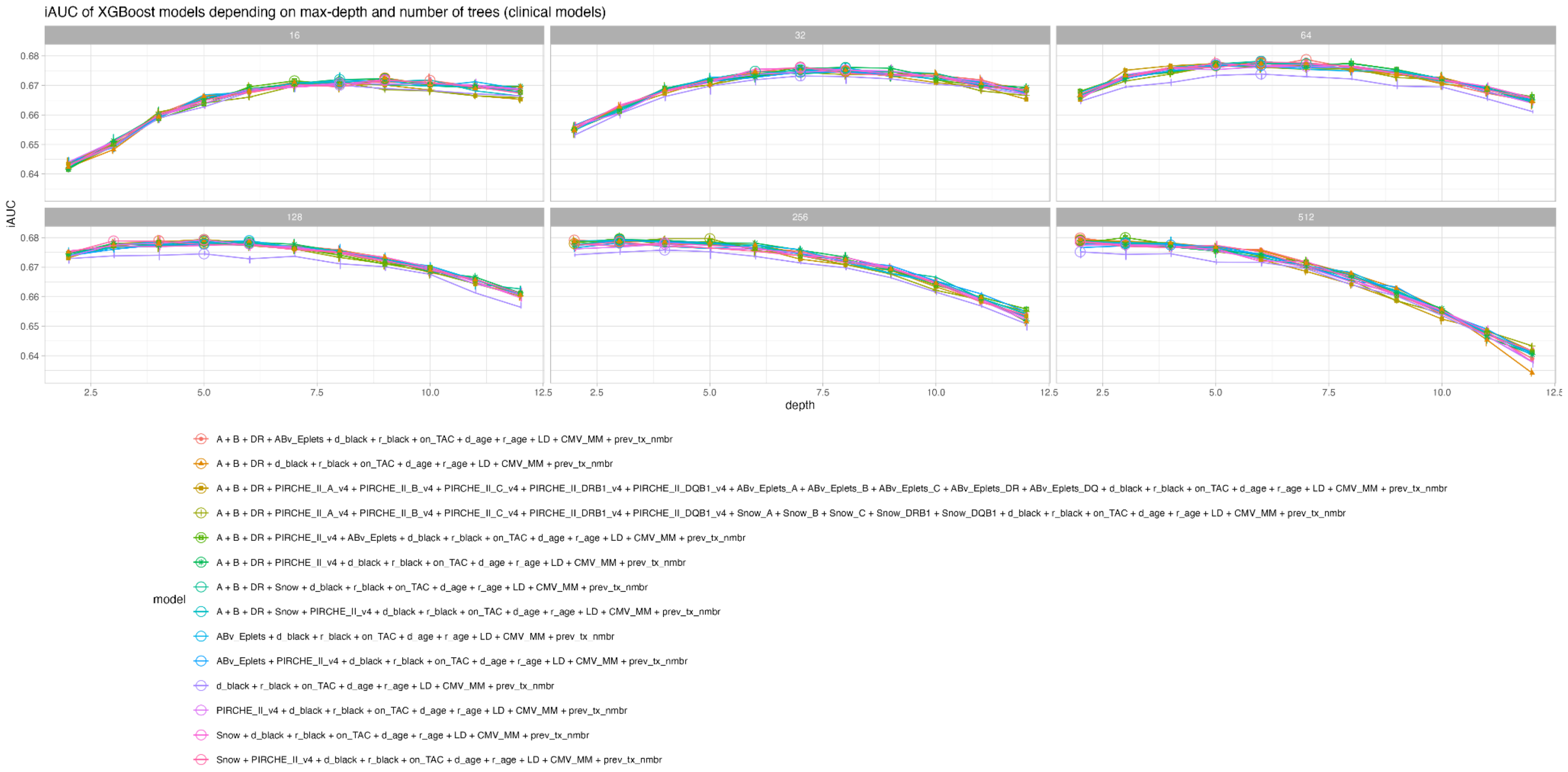

Supplementary Fig 16: Integrated AUC index (y axis) of repeated XGBoost models of clinical parameters considering maximum model depth (x axis) and number of trees per model (panels). Points indicate the respective median C index with the error bars indicating the first and third quartiles. Circles around points indicate the respective models maximum median value per panel.

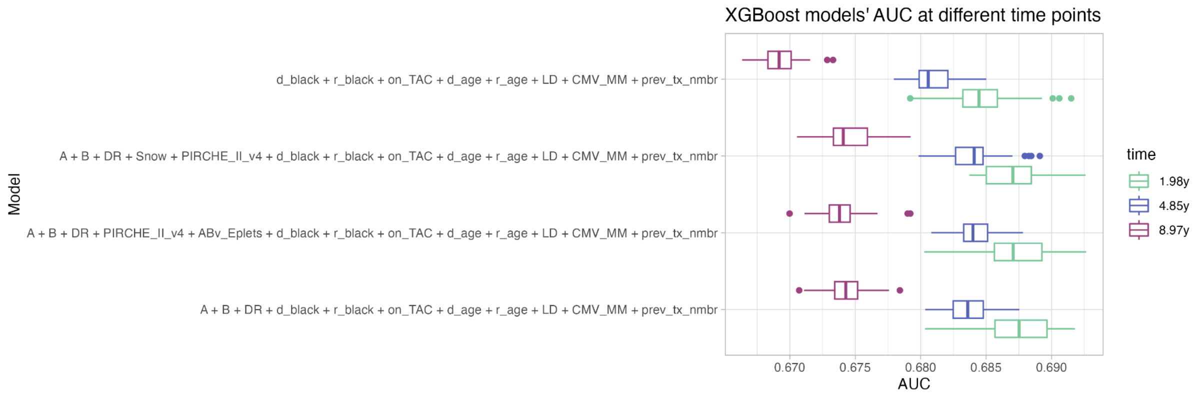

Supplementary Fig 17: Distribution of repeated XGBoost models' AUC at 1.98 years, 4.85 years and 8.97 years (corresponding to the 25th, 50th and 75th percentiles of the observation period) indicate higher performance of the histocompatibility-augmented models at all tested time points with a slight advantage of molecular matching-containing models in short- and mid-term prediction.

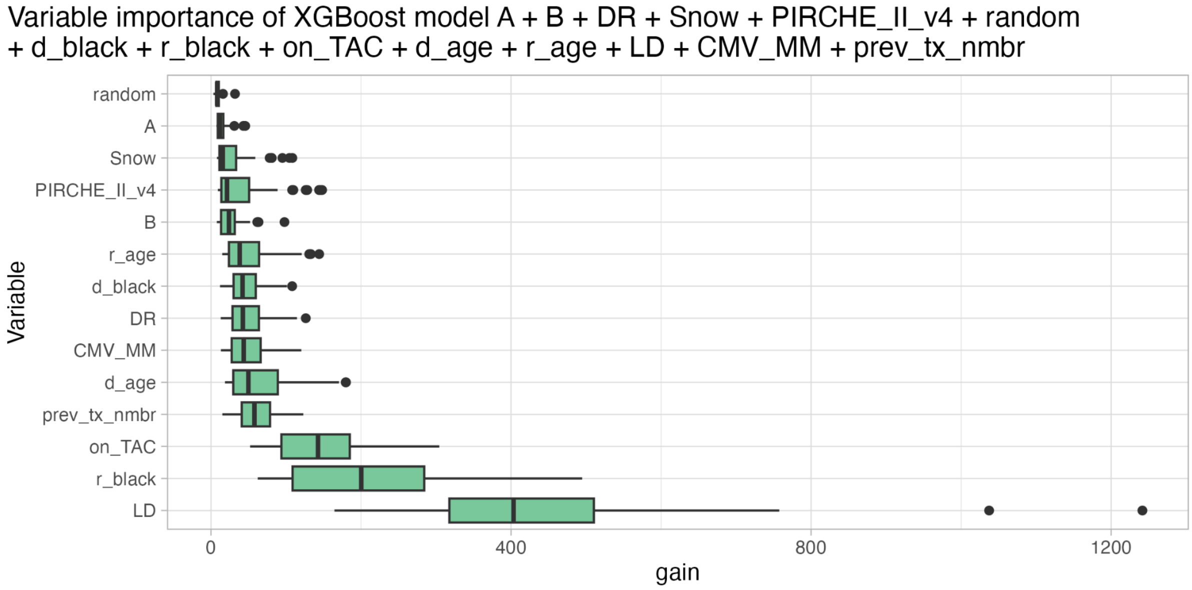

Supplementary Fig 18: Variable importance based on gain metric (higher is better) of XGBoost model considering HLA-A, -B, -DR matching, Snow, PIRCHE-II (PIRCHE_II_v4), donor being black (d_black), recipient being black (r_black), Tacrolimus maintenance (on_TAC), donor age (d_age), recipient age (r_age), living donor transplantation (LD, most important), CMV mismatch (CMV_MM), number of previous transplantations (prev_tx_nmbr) and a random variable (least important). Even though HLA-A matching is of low overall importance, it still outperforms a variable of random values artificially included into the model.

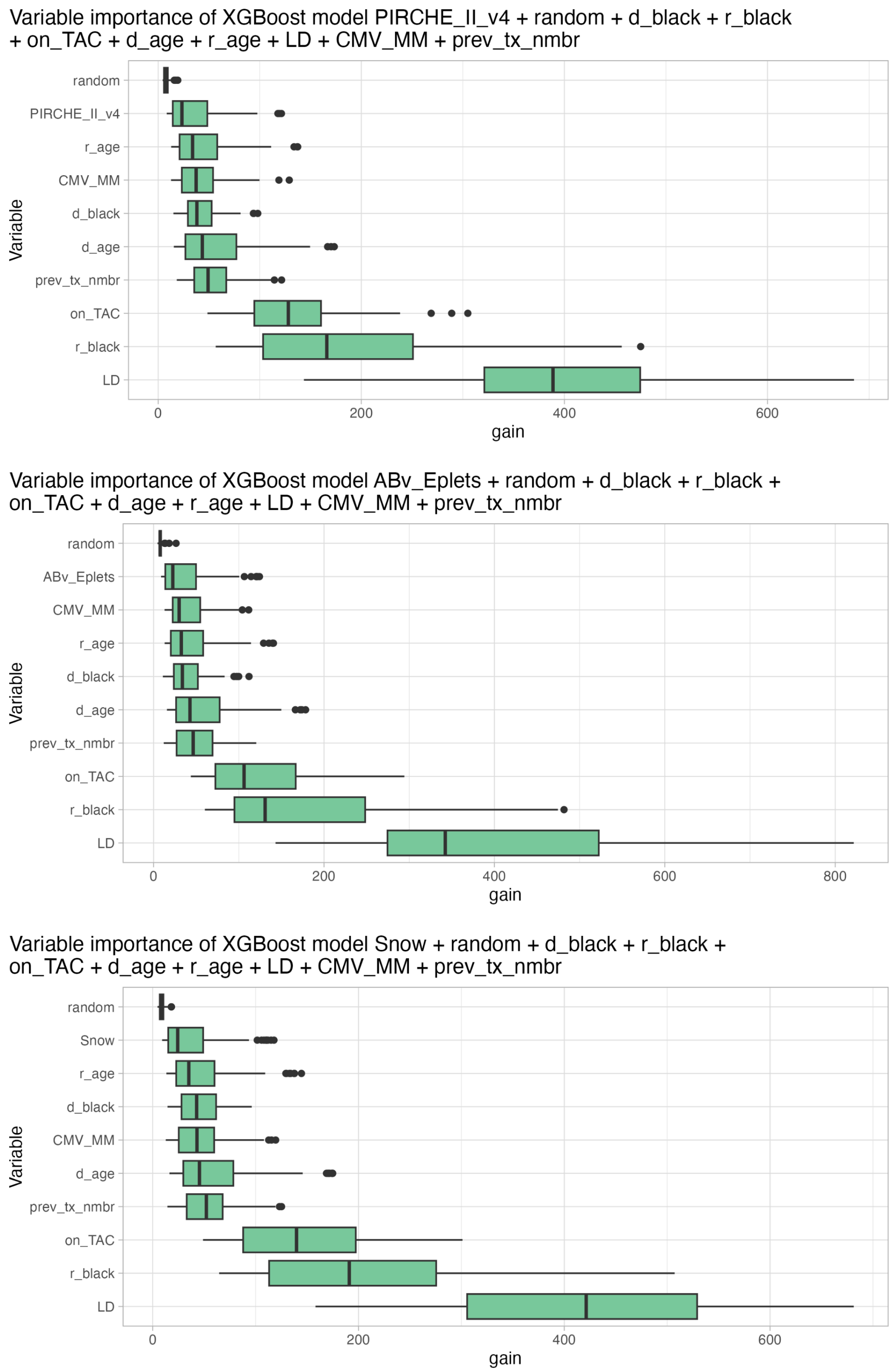

Supplementary Fig 19: Variable importance based on gain metric (higher is better) of XGBoost model considering clinical parameters (donor being black (d_black), recipient being black (r_black), Tacrolimus maintenance (on_TAC), donor age (d_age), recipient age (r_age), living donor transplantation (LD, most important), CMV mismatch (CMV_MM), number of previous transplantations (prev_tx_nmbr), a random variable (least important), and (A) PIRCHE-II (PIRCHE_II_v4), (B) antibody-verified Eplets (ABv_Eplets), and (C) Snow. Molecular matching is ranked having the least overall importance, yet it is in the same order of magnitude as recipient age and clearly outperforms a random variable artificially included into the model.

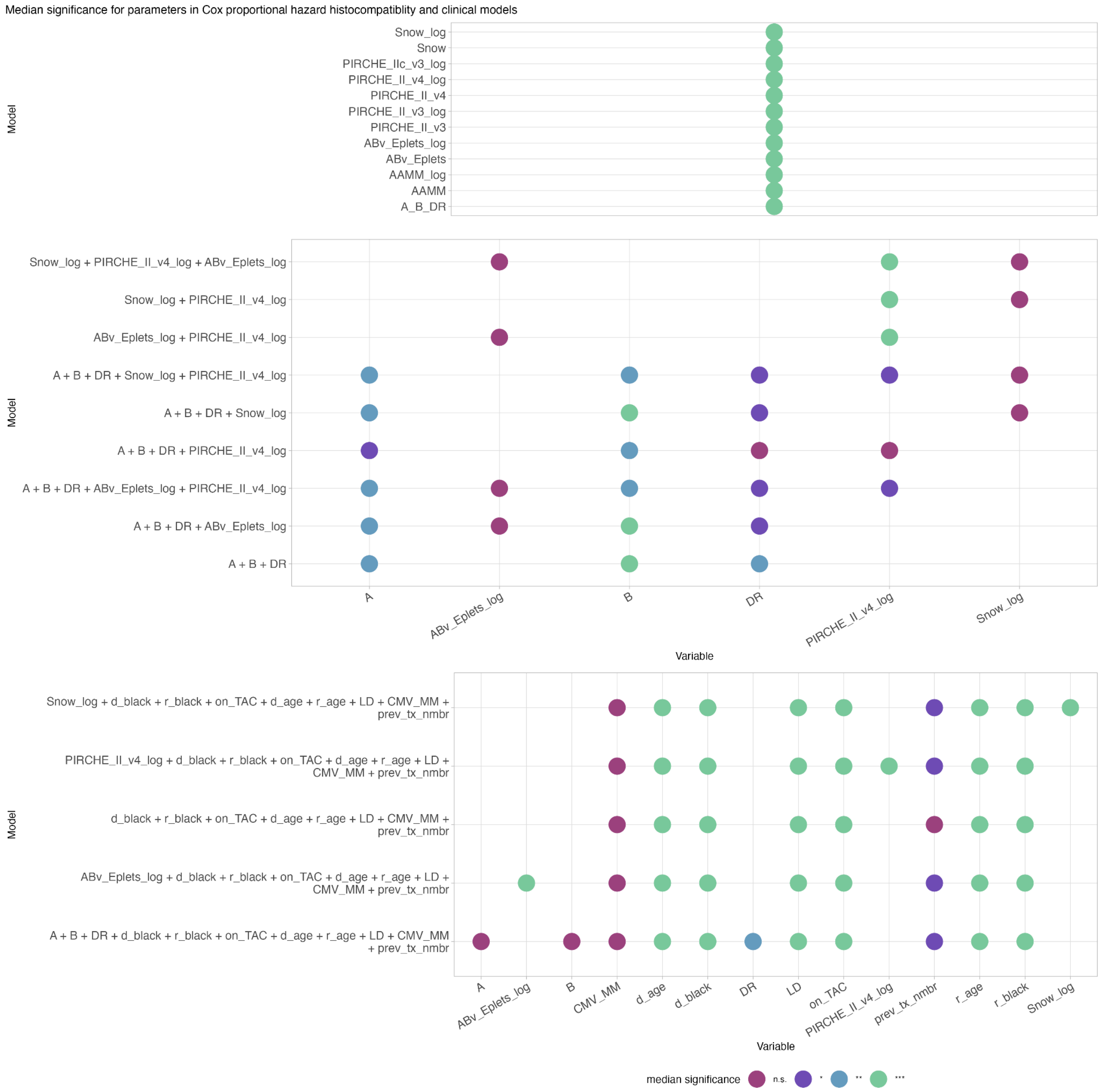

Supplementary Fig 20: Median significance levels of repeated Cox proportional hazard models considering the subgroup of patients transplanted within the first two years after implementation of the new 2014 Kidney Allocation System (KAS). n.s.: p >= 0.5, *: p < 0.05, **: p < 0.01, ***: p < 0.001

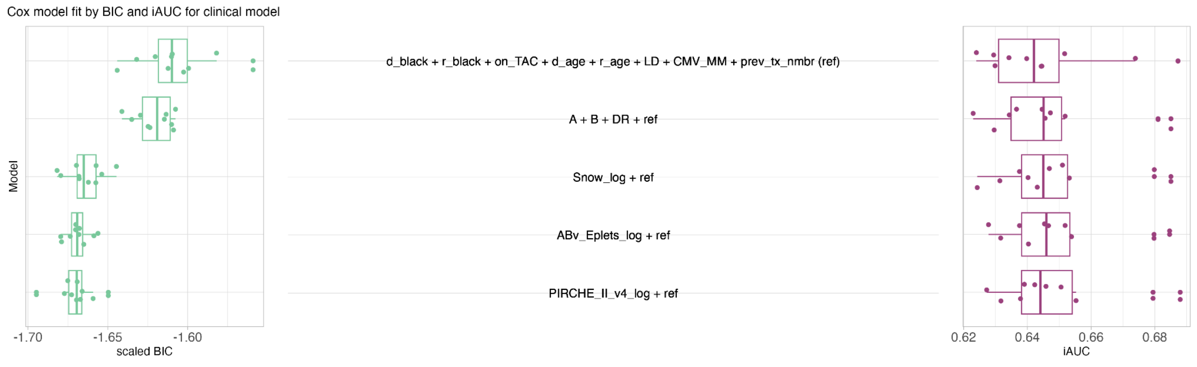

Supplementary Fig 21: Extended butterfly plot of scaled BIC (green, left, lower is better) and mAUC (purple, right, higher is better) of Cox proportional hazards models considering histocompatibility metrics predicting graft survival using the subgroup of patients transplanted within the first two years after implementation of the new 2014 Kidney Allocation System (KAS). Boxplots depict the median (horizontal line), first to third quartile (box); the highest and lowest values within 1.5× IQR (whiskers) and outliers (circles), respectively.

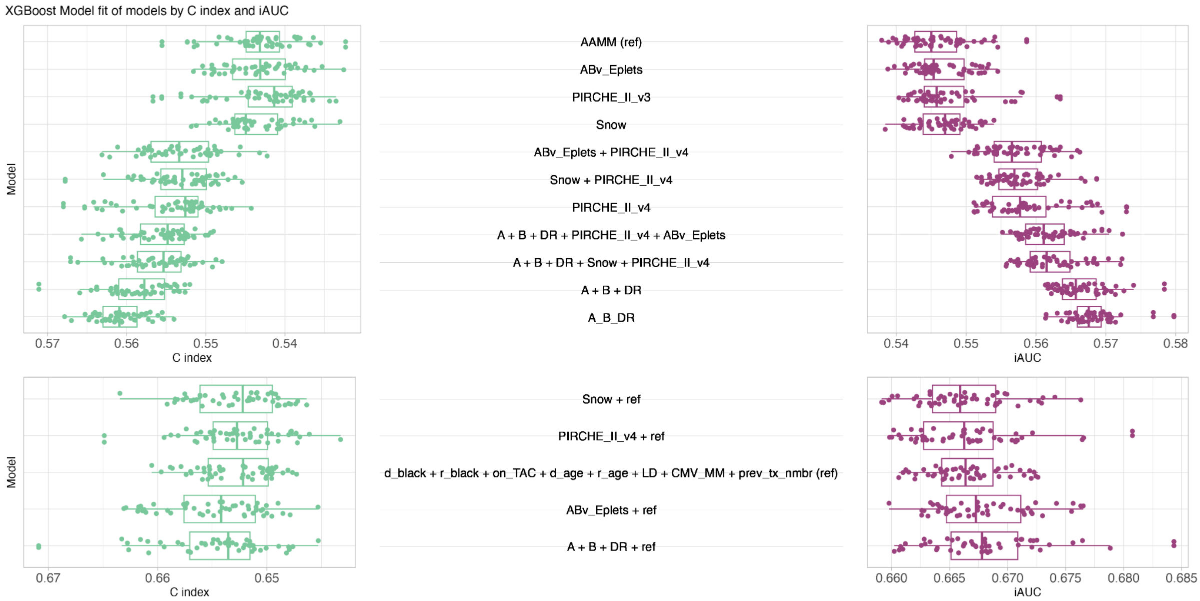

Supplementary Fig 22: Butterfly plot of ten best XGBoost models' performance in terms of C index (blue, left, larger is better) and iAUC (pink, right, larger is better) considering the subgroup of patients transplanted within the first two years after implementation of the new 2014 Kidney Allocation System (KAS). Top panel considers models only consisting of histocompatibility metrics, bottom panel considers models of known clinical risk factors in conjunction with histocompatibility metrics.. Boxplots depict the median (horizontal line), first to third quartile (box); the highest and lowest values within 1.5× IQR (whiskers) and outliers (circles), respectively.

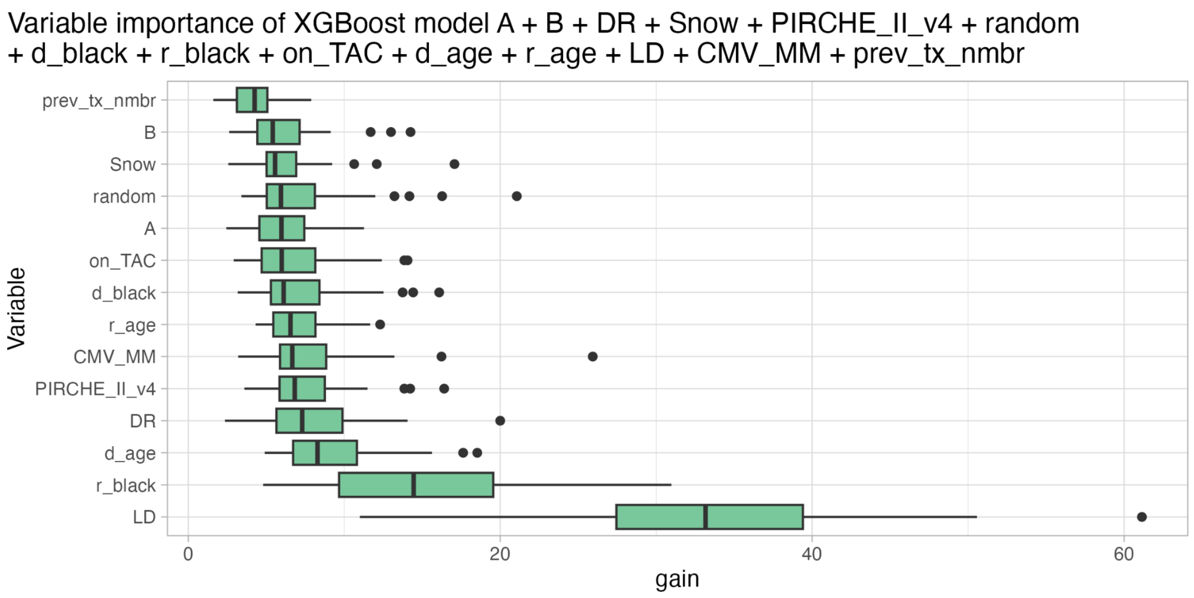

Supplementary Fig 23: Variable importance based on gain metric (higher is better) accounting for the subgroup of patients transplanted within the first two years after implementation of the new 2014 Kidney Allocation System (KAS) considering an XGBoost model ensemble of HLA-A, -B, -DR matching, Snow, PIRCHE-II (PIRCHE_II_v4), donor being black (d_black), recipient being black (r_black), Tacrolimus maintenance (on_TAC), donor age (d_age), recipient age (r_age), living donor transplantation (LD, most important), CMV mismatch (CMV_MM), number of previous transplantations (prev_tx_nmbr, least important) and a random variable. Notably, the number of previous transplantations is of lower importance than a random value (random) artificially included into the model.

Supplementary Table 1: Mapping of SRTR-reported race and ethnicity values to populations reported by Maiers et al [1].

SRTR race	SRTR ethnicity	Mapped to NMDP 2007 haplotype population
WHITE	LATINO	HIS
ASIAN	LATINO	HIS
BLACK	LATINO	HIS
MULTI	LATINO	HIS
NATIVE	LATINO	HIS
PACIFIC	LATINO	HIS
WHITE	N/LATINO	EUR
ASIAN	N/LATINO	API
BLACK	N/LATINO	AFA
MULTI	N/LATINO	EUR
NATIVE	N/LATINO	EUR
PACIFIC	N/LATINO	API

Supplementary Table 2: Included external R and python libraries.

Library	Environment	Reference
ggplot2	R	[2]
data.table	R	[3]
caret	R	[4]
tidyr	R	[5]
tidyverse	R	[6]
ggstatsplot	R	[7]
dplyr	R	[8]
reshape2	R	[9]
grid	R	[10]
gridExtra	R	[11]
akima	R	[12]
rgl	R	[13]
viridis	R	[14]
scales	R	[15]
foreach	R	[16]
doParallel	R	[17]
doSNOW	R	[18]
haven	R	[19]
survminer	R	[20]
survival	R	[21]
ROCR	R	[22]
pROC	R	[23]
MuMIn	R	[24]
survAUC	R	[25]
survivalROC	R	[26]
numpy	python	[27]
xgboost	python	[28,29]

pandas	python	[30]
scikit-learn	python	[31]
scikit-survival	python	[32–34]

Bibliography

1. Maiers M, Gragert L, Klitz W. High-resolution HLA alleles and haplotypes in the United States population. Human Immunology. 2007 Sep;68(9):779–88.
2. Wickham H. ggplot2: Elegant Graphics for Data Analysis [Internet]. Springer-Verlag New York; 2016. Available from: https://ggplot2.tidyverse.org
3. Dowle M, Srinivasan A. data.table: Extension of `data.frame` [Internet]. 2023. Available from: https://CRAN.R-project.org/package=data.table
4. Kuhn, Max. Building Predictive Models in R Using the caret Package. Journal of Statistical Software. 2008;28(5):1–26.
5. Wickham H, Vaughan D, Girlich M. tidyr: Tidy Messy Data [Internet]. 2023. Available from: https://CRAN.R-project.org/package=tidyr
6. Wickham H, Averick M, Bryan J, Chang W, McGowan LD, François R, Grolemund G, Hayes A, Henry L, Hester J, Kuhn M, Pedersen TL, Miller E, Bache SM, Müller K, Ooms J, Robinson D, Seidel DP, Spinu V, Takahashi K, Vaughan D, Wilke C, Woo K, Yutani H. Welcome to the tidyverse. Journal of Open Source Software. 2019;4(43):1686.
7. Patil I. Visualizations with statistical details: The "ggstatsplot" approach. Journal of Open Source Software. 2021;6(61):3167.
8. Wickham H, François R, Henry L, Müller K, Vaughan D. dplyr: A Grammar of Data Manipulation [Internet]. 2023. Available from: https://CRAN.R-project.org/package=dplyr
9. Wickham H. Reshaping Data with the reshape Package. Journal of Statistical Software. 2007;21(12):1–20.
10. R Core Team. R: A Language and Environment for Statistical Computing [Internet]. Vienna, Austria: R Foundation for Statistical Computing; 2022. Available from: https://www.R-project.org/
11. Auguie B. gridExtra: Miscellaneous Functions for "Grid" Graphics [Internet]. 2017. Available from: https://CRAN.R-project.org/package=gridExtra
12. Akima H, Gebhardt A. akima: Interpolation of Irregularly and Regularly Spaced Data [Internet]. 2022. Available from: https://CRAN.R-project.org/package=akima
13. Murdoch D, Adler D. rgl: 3D Visualization Using OpenGL [Internet]. 2023. Available from: https://CRAN.R-project.org/package=rgl
14. Garnier, Simon, Ross, Noam, Rudis, Robert, Camargo, Pedro A, Sciaini, Marco, Scherer, Cédric. viridis - Colorblind-Friendly Color Maps for R [Internet]. 2021. Available from: https://sjmgarnier.github.io/viridis/
15. Wickham H, Seidel D. scales: Scale Functions for Visualization [Internet]. 2022. Available from: https://CRAN.R-project.org/package=scales
16. Microsoft, Weston S. foreach: Provides Foreach Looping Construct [Internet]. 2022. Available from: https://CRAN.R-project.org/package=foreach
17. Corporation M, Weston S. doParallel: Foreach Parallel Adaptor for the "parallel" Package [Internet]. 2022. Available from: https://CRAN.R-project.org/package=doParallel
18. Corporation M, Weston S. doSNOW: Foreach Parallel Adaptor for the "snow" Package [Internet]. 2022. Available from: https://CRAN.R-project.org/package=doSNOW
19. Wickham H, Miller E, Smith D. haven: Import and Export "SPSS", "Stata" and "SAS" Files [Internet]. 2023. Available from: https://CRAN.R-project.org/package=haven
20. Kassambara A, Kosinski M, Biecek P. survminer: Drawing Survival Curves using

"ggplot2" [Internet]. 2021. Available from: https://CRAN.R-project.org/package=survminer
21. Terry M. Therneau, Patricia M. Grambsch. Modeling Survival Data: Extending the Cox Model. New York: Springer; 2000.
22. Sing T, Sander O, Beerenwinkel N, Lengauer T. ROCR: visualizing classifier performance in R. Bioinformatics. 2005;21(20):7881.
23. Robin X, Turck N, Hainard A, Tiberti N, Lisacek F, Sanchez JC, Müller M. pROC: an open-source package for R and S+ to analyze and compare ROC curves. BMC Bioinformatics. 2011;12:77.
24. Bartoń K. MuMIn: Multi-Model Inference [Internet]. 2023. Available from: https://CRAN.R-project.org/package=MuMIn
25. Potapov S, Adler W, Schmid M. survAUC: Estimators of Prediction Accuracy for Time-to-Event Data [Internet]. 2023. Available from: https://CRAN.R-project.org/package=survAUC
26. Heagerty PJ, Saha-Chaudhuri packaging by P. survivalROC: Time-Dependent ROC Curve Estimation from Censored Survival Data [Internet]. 2022. Available from: https://CRAN.R-project.org/package=survivalROC
27. Harris CR, Millman KJ, Van Der Walt SJ, Gommers R, Virtanen P, Cournapeau D, Wieser E, Taylor J, Berg S, Smith NJ, Kern R, Picus M, Hoyer S, Van Kerkwijk MH, Brett M, Haldane A, Del Río JF, Wiebe M, Peterson P, Gérard-Marchant P, Sheppard K, Reddy T, Weckesser W, Abbasi H, Gohlke C, Oliphant TE. Array programming with NumPy. Nature. 2020 Sep 17;585(7825):357–62.
28. xgboost developers. XGBoost Documentation [Internet]. 2024. Available from: https://xgboost.readthedocs.io
29. Chen T, Guestrin C. XGBoost: A Scalable Tree Boosting System. In: Proceedings of the 22nd ACM SIGKDD International Conference on Knowledge Discovery and Data Mining [Internet]. San Francisco California USA: ACM; 2016 [cited 2024 Feb 2]. p. 785–94. Available from: https://dl.acm.org/doi/10.1145/2939672.2939785
30. McKinney W. Data Structures for Statistical Computing in Python. In Austin, Texas; 2010 [cited 2024 Feb 2]. p. 56–61. Available from: https://conference.scipy.org/proceedings/scipy2010/mckinney.html
31. Pedregosa F, Varoquaux G, Gramfort A, Michel V, Thirion B, Grisel O, Blondel M, Prettenhofer P, Weiss R, Dubourg V, Vanderplas J, Passos A, Cournapeau D, Brucher M, Perrot M, Duchesnay É. Scikit-learn: Machine Learning in Python. J Mach Learn Res. 2011 Nov;12(null):2825–30.
32. Pölsterl S, Navab N, Katouzian A. Fast Training of Support Vector Machines for Survival Analysis. In: Appice A, Rodrigues PP, Santos Costa V, Gama J, Jorge A, Soares C, editors. Machine Learning and Knowledge Discovery in Databases. 2015. p. 243–59. (Lecture Notes in Computer Science).
33. Pölsterl S, Navab N, Katouzian A. An Efficient Training Algorithm for Kernel Survival Support Vector Machines. In: 3rd Workshop on Machine Learning in Life Sciences [Internet]. 2016. Available from: https://arxiv.org/abs/1611.07054
34. Pölsterl S, Pankaj G, Wang L, Conjeti S, Katouzian A, Navab N. Heterogeneous Ensembles for Predicting Survival of Metastatic, Castrate-Resistant Prostate Cancer Patients. F1000Research. 2016 Nov;5(2676).
